# Effect of Dimeric Disintegrins Isolated from *Vipera lebetina obtusa* Venom on Glioblastoma Cellular Responses

**DOI:** 10.3390/cancers15194805

**Published:** 2023-09-29

**Authors:** Anna Galicka, Łukasz Szoka, Iwona Radziejewska, Cezary Marcinkiewicz

**Affiliations:** 1 Department of Medical Chemistry, Medical University of Bialystok, Mickiewicza 2A, 15-222 Bialystok, Poland; iwona@umb.edu.pl; 2 Department of Medicinal Chemistry, Medical University of Bialystok, Mickiewicza 2D, 15-222 Bialystok, Poland; lukasz.szoka@umb.edu.pl; 3 Department of Bioengineering, Temple University CoE, Philadelphia, PA 19406, USA

**Keywords:** glioblastoma, α5β1 integrin, VLO4 disintegrin, adhesion, spreading, survival, migration, apoptosis, MMP-2/MMP-9

## Abstract

**Simple Summary:**

Integrins play an important role in the development and spread of cancers, including glioblastoma (GBM); therefore, blocking them should be an effective cancer treatment. Our research aims to use the RGD homodimeric disintegrin VLO4, isolated from *Vipera lebetina obtusa* venom, to target the α5β1 integrin, which is a key regulator of GBM cell migration and invasion. This study shows its effect on the adhesion, spreading, migration, and survival of the LBC3, LN18, and LN229 cell lines. The presented results suggest that VLO4 may be a structural platform to design therapeutics for gliomas, which may be used alone or in combination with other integrin blockers such as inhibitors of α9β1 integrin (e.g., structures based on MLD disintegrin, VLO5).

**Abstract:**

Integrins play a fundamental role in the migration and invasiveness of glioblastoma (GBM) cells, making them suitable targets for innovative cancer therapy. The aim of this study was to evaluate the effect of the RGD homodimeric disintegrin VLO4, isolated from *Vipera lebetina obtusa* venom, on the adhesion, spreading, migration, and survival of LBC3, LN18, and LN229 cell lines. This disintegrin, as a potent antagonist for α5β1 integrin, showed pro-adhesive properties for these cell lines, the highest for LN229 and the lowest for LBC3. Glioblastoma cells displayed significant differences in the spreading on the immobilized VLO4 and the natural α5β1 integrin ligand, fibronectin. Solubilized VLO4 showed different cytotoxicity and pro-apoptotic properties among tested cell lines, with the highest against LN18 and none against LN229. Moreover, VLO4 revealed an inhibitory effect on the migration of LBC3 and LN18 cell lines, in contrast to LN229 cells, which were not sensitive to this disintegrin. However, LN229 migration was impaired by VLO5, a disintegrin antagonistic to integrin α9β1, used in combination with VLO4. A possible mechanism of action of VLO4 may be related to the downregulation of α5β1 integrin subunit expression, as revealed by Western blot. VLO4 also inhibited cell proliferation and induced caspase-dependent apoptosis in LBC3 and LN18 cell lines. These results indicate that targeting α5β1 integrin by related VLO4 compounds may be useful in the development of integrin-targeted therapy for glioblastoma.

## 1. Introduction

According to the World Health Organization (WHO) classification (2021) of the Central Nervous System (CNS) tumors, there are four main groups of diffuse gliomas: adult-type diffuse gliomas, pediatric-type diffuse low-grade gliomas, pediatric-type diffuse high-grade gliomas, and circumscribed astrocytic gliomas [1]. Glioblastoma (GBM), classified as CNS WHO grade 4, constitutes adult isocitrate dehydrogenase (IDH)-wild-type diffuse astrocytic glioma and is characterized by microvascular proliferation and/or necrosis, mutation of telomerase reverse transcriptase (*TERT*) promotor, amplification of epidermal growth factor receptor (*EGFR*) gene, or changes in +7/–10 chromosome copy number [1]. GBM is the most common and aggressive of the primary brain tumors, accounting for 57% of all gliomas and 48% of primary central nervous system (CNS) tumors [2,3].

Glioblastoma is a biologically heterogeneous and highly complex neoplasia that is very difficult to treat. Despite advances in therapy research, its management still relies on maximum safe resection, radiotherapy, and Temozolomide (TMZ) chemotherapy [2,4,5], while multi-resistance mechanisms, immunosuppressive microenvironment, and high tumor invasiveness constitute limitations to standard treatment [6,7,8]. TMZ treatment is usually more effective in patients with promoter methylation of O^6^-methylguanine DNA methyltransferase (MGMT), while the effect of this drug in patients with tumors with unmethylated MGMT promoters is small. However, it turns out that even in the case of hypermethylated MGMT in GBM cells, this enzyme is highly expressed regardless of the promoter, thus developing resistance to TMZ treatment [8]. All glioblastomas eventually progress or recur, and there is no standard treatment for recurrent GBM. The most commonly used treatment for recurrent GBM is another alkylating agent, Lomustine; however, like TMZ, it is only effective in patients with MGMT promoter methylation [8,9]. Bevacizumab, a vascular endothelial growth factor (VEGF)-A targeting monoclonal antibody, can only prolong progression-free survival in recurrent GBM, while combination therapy, although it reduces the aggressiveness of the tumor in the initial phase, does not significantly affect the course of the disease [8,9,10].

Recently, various strategies have been used alone or in combination in preclinical studies and several clinical trials. These include, among others, immunotherapy approaches (Chimeric antigen receptor (CAR) T cell therapy, immune checkpoint blockade, oncolytic virus, cytokine- and vaccine-based therapies), Tumor Treating Fields (TTF) therapy, or gene editing/silencing technology [2,5,6,7,10,11,12]. Most of them turned out to be insufficiently effective, considering the average patient survival time, which is 16–24 months after diagnosis, even with aggressive therapy [13].

Although this tumor rarely metastasizes to peripheral organs, the poor prognosis of patients is caused by its aggressive local growth pattern and the massive and diffuse infiltration of the surrounding normal brain tissue [6,7,14]. The infiltration is specifically controlled by the interactions between tumor cells and the surrounding brain microenvironment [6,7], in which integrins play a fundamental role and therefore have become an attractive molecular target for therapeutic intervention in GBM [15,16,17,18].

Integrins are the major cell surface receptors that play a key role in adhesion of cells to extracellular matrix (ECM) proteins and cell-cell interaction [15,16,17]. They participate in both inside-out and outside-in signal transduction processes and regulate cellular responses such as adhesion, motility, migration, invasion, protease secretion, and angiogenesis [19,20]. Based on the binding specificity of the ligands, they can be divided into integrins that bind the collagen, laminin, or arginine-glycine-aspartic acid (RGD) motif, and leukocyte-specific receptors. In their active conformation, integrins contain one α and one β subunit, and in mammalian cells, 18 α and 8 β subunits form 24 different heterodimer combinations [20]. Despite having a significant role in normal cell physiology, integrins have also been characterized as stimulators of pathological outcomes, especially cancer [15,16,17,18,21]. Many of them have been shown to participate in the regulation of tumor growth and angiogenesis. The malignant transformation of the cell is usually associated with rearrangements of the plasma membrane environment, including the expression of integrins. In the case of GBM, the expression of α2β1, α3β1, α5β1, α6β1, α7β1, α10β1, and αvβ3 integrins is strongly up-regulated [16,17,18,22,23,24,25], while α9β1 integrin is undetectable in normal brain tissue and is expressed in most glioblastomas [26,27].

The α5β1 integrin was characterized as a major fibronectin receptor, which belongs to the RGD-dependent family. This integrin is overexpressed in high-grade gliomas and associated with a poorer prognosis for patients [28,29,30]. There are reports that the use of antagonists of this integrin reduces the proliferation and clonogenicity of GBM cells [31,32] and may sensitize glioblastoma cells to chemotherapeutic drugs [29,33]. Inhibition of its expression delayed tumor growth in a mouse model of glioblastoma [34]. These studies suggest that the α5β1 integrin may be a therapeutic target for this cancer; however, there is a need to increase research into the search for new antagonists.

Disintegrins are a family of low-molecular-weight proteins isolated from the venom of various vipers that selectively bind certain integrins and can act as competitive inhibitors of natural ligands. Structurally, they were divided into monomeric and dimeric molecules, generally displaying in their active sites RGD, MLD, or KTS active motifs. Many studies show that they have great potential in anti-cancer therapy [35,36,37].

VLO4 was isolated from *Vipera lebetina obtusa* venom and belongs to the homodimeric disintegrins family. It expresses an RGD motif and was characterized as a potent and quite selective antagonist of α5β1 integrin [37]. In this study, we investigated the effect of this disintegrin on the cellular responses of the human glioblastoma cell lines LBC3, LN18, and LN229.

## 2. Materials and Methods

### 2.1. Chemicals

Dulbecco’s minimal essential medium (DMEM), fetal bovine serum (FBS), and phosphate-buffered saline (PBS) were obtained from Gibco (Thermo Fisher Scientific, Waltham, MA, USA); glutamine, penicillin, and streptomycin were purchased from Quality Biologicals Inc. (Gaithersburg, MD, USA). Radioimmunoprecipitation assay (RIPA) buffer, protease inhibitor cocktail (P8340), and [3-(4,5-dimethylthiazol-2-yl)-2,5-diphenyltetrazolium bromide] (MTT), gelatin, fibronectin, and type I collagen were provided by Sigma-Aldrich Corp. (St. Louis, MO, USA).

### 2.2. Cell Lines, Antibodies and Snake Venom Disintegrins

Glioblastoma cell lines (LN18 and LN229) were purchased from ATCC (Manassas, VA, USA). The LBC3 cell line was developed from GBM tissue after surgical resection and is characterized by a mutation in the p53 protein [38]. Mouse monoclonal antibodies: anti-α2 and anti-β1 integrin subunits were purchased from Santa Cruz Biotechnology Inc. (Santa Cruz, CA, USA); anti-α5 was purchased from Becton-Dickinson (Franklin Lakes, NJ, USA). Polyclonal serum against the a9 subunit of the integrin cytoplasmic domain was developed commercially in rabbits (Chemicon, Temecula, CA, USA); rabbit monoclonal anti-Bax, anti-Bcl2, anti-Bcl-xL, and anti-caspase-3 (cleaved) were purchased from Cell Signaling Technology (Beverley, MA, USA); rabbit monoclonal anti-β-actin was purchased from Sigma-Aldrich Corp. (St. Louis, MO, USA). The horseradish peroxidase-conjugated secondary antibody, anti-mouse immunoglobulin G (IgG) (whole molecule), was obtained from Sigma-Aldrich Corp. (St. Louis, MO, USA), and anti-rabbit IgG was purchased from Cell Signaling Technology (Beverley, MA, USA). VLO4 and VLO5 disintegrins were purified from the venom of *Vipera lebetina obtusa* (Latoxan, Valence, France) using two steps of reverse-phase high-performance liquid chromatography [39].

### 2.3. Cell Line Culture and Treatment

Cells were cultured in DMEM supplemented with 10% FBS, 2 mM glutamine, penicillin (50 U/mL), and streptomycin (50 µg/mL). They were maintained in a humidified 5% CO_2_/air incubator at 37 °C. For most experiments, cells were grown to 90% confluence and treated with VLO4/VLO5 disintegrins for 24 h.

### 2.4. Cytotoxicity of VLO4 Disintegrin Assay

The cytotoxicity of VLO4/VLO5 disintegrins towards three glioblastoma cell lines was measured by the colorimetric MTT assay. Approximately 1 × 10^4^ cells per well (in 24-well plates) were treated with VLO4 or VLO5 for 24 h. Then, cells were washed with PBS, and MTT (0.5 mg/mL) was added for 4 h. After that, the MTT solution was removed, and 1 mL of 0.1 M HCl, dissolved in absolute isopropanol, was added. The plate was gently shaken on a plate shaker (BioSan, Riga, Latvia), and the absorbance at 570 nm was measured using a microplate reader (TECAN, Männedorf, Switzerland).

### 2.5. Cell Migration/Scratch Assay

To measure the effects of disintegrins on cell migration, a wound healing/scratch assay was performed. Cells (5 × 10^5^/mL per well) were seeded in a 6-well plate and allowed to grow to confluence in DMEM with 10% FBS. The cultured medium was then replaced with medium containing 2% FBS (to reduce proliferation of cells), and a scratch in the cell monolayer was made with a sterile 200 µL pipette tip. After extensive washing with medium to remove cell debris, cells were incubated with VLO4 or VLO5 disintegrins for 24 h; the untreated ones served as controls. Images were analyzed using an inverted optical microscope at 40× magnification (Nikon; Minato, Tokyo, Japan). Migration was determined by measurements of the width of the scratch for each cell line and calculated using the equation [(S − F)/S] × 100, where S is the distance (mm) of the cell edge at 0 h and F is the distance (mm) of the cell edge at 24 h.

### 2.6. Cell Adhesion Assays

The Cell adhesion assay was performed according to the procedure described previously [38]. Briefly, VLO4 at the indicated concentrations, as well as fibronectin or collagen type I at concentrations of 10 μg/mL each, were immobilized on the 96-well plate in PBS by overnight incubation at 4 °C. Wells were blocked with 1% BSA in Hanks’ balanced salt solution (HBSS) by incubation for 1 h at room temperature. Cells were detached from the tissue culture plate by trypsinization and labeled with 5-chloromethylfluorescein diacetate (CMFDA) by incubation at 37 °C for 30 min. CMFDA-labeled cells were added to the wells in the amount of 1 × 10^5^ and suspended in 100 μL of HBSS containing 1% BSA. The plate was incubated at 37 °C for 30 min, unattached cells were removed by vacuum aspiration, and wells were washed 3× with HBSS containing 1% BSA. Attached cells were solubilized by 0.5% Triton X-100, and plates were read using a fluorescence plate reader (BioTek, Instruments, Vinooski, VT, USA) with an excitation wavelength of 485 nm and an emission wavelength of 530 nm. The number of adhered cells was calculated from a standard curve prepared from known amounts of cells on the same plate.

### 2.7. Cell Spreading

Evaluation of cell spreading area was performed on 8-well glass chamber slides with immobilized fibronectin (10 μg/mL) or VLO4 (10 μg/mL) by overnight incubation at 4 °C in PBS. Slides were blocked with 1% BSA, and cells (5 × 10^4^/mL) were added in serum-free DMEM. Slides were incubated at 37 °C for 1 h and then fixed with 4% paraformaldehyde. Cells were washed 3× with PBS and permeabilized by incubation with 0.4% Triton X-100 on the ice for 5 min, and mounting buffer containing TRITC-phalloidin and DAPI was added. Slides were analyzed using a fluorescence microscope (Olympus IX81, Olympus, Tokyo, Japan) with a 40× oil objective.

### 2.8. Western Blot

Cells were lysed in RIPA buffer (Sigma-Aldrich Corp., St. Louis, MO, USA) with an added cocktail of protease inhibitors (P8340) (Sigma-Aldrich Corp., St. Louis, MO, USA). The BCA Protein Assay Kit (Pierce, Rockford, IL, USA) was used to measure the concentration of total protein in cell lysates. Approximately 20 or 40 µg of protein was resolved in SDS-polyacrylamide gels and transferred onto Immobilon-P Transfer membranes (Merck Millipore Ltd., Tullagreen, Carrigtwohill, County Cork, Ireland). After blocking with non-fat dried milk (5% *w*/*v*) in 50 mM Tris-HCl, pH 7.5, 500 mM NaCl, and 0.05% (*v*/*v*) Tween 20 (TBS-T) for 1 h at room temperature, membranes were rinsed in TBS-T before incubation with primary antibodies. The membranes were usually cut at a specific height based on the molecular weight marker and separately developed to detect two different proteins in the same samples separated in the same gel or to detect the tested protein and the reference protein (β-actin) simultaneously. After overnight incubation at 4 °C with primary antibodies (1:1000), the secondary antibody (1:2000), conjugated with horseradish peroxidase, was added for 1 h of incubation. The proteins were visualized in the BioSpectrum Imaging System (Ultra-Violet Products, Ltd., Cambridge, UK) using Westar Supernova Chemiluminescent Substrate (Cyanagen, Bologna, Italy). Densitometric measurement (G:BOX, Syngene, Cambridge, UK) was used to analyze the expression of proteins relative to β-actin.

### 2.9. Immunocytochemistry

Cells were seeded in a glass chamber and cultured in DMEM containing 10% FBS until 80–90% confluency. FITC-VLO4 (1 mg/mL) or FITC-VLO5 (1 mg/mL) was added to the same media, and incubation was performed for 1 h at 37 °C. Cells were fixed with 4% paraformaldehyde by incubation for 1 h at room temperature. Cells were washed 3× with PBS and permeabilized by incubation with 0.4% Triton X-100 on the ice for 5 min. Slides were blocked with 1% BSA in PBS by incubation for 1 h at room temperature, and mounting buffer containing TRITC-phalloidin and DAPI was added. Slides were analyzed using a fluorescence microscope (Olympus IX81, Olympus, Tokyo, Japan) with a 40× oil objective.

### 2.10. Zymography for the Determination of MMPs Activity

For the determination of MMPs activity, using a sensitive zymography method, cells were incubated in serum-free DMEM because these enzymes also occur in serum and may falsify this assay. The same amount of media proteins were electrophoresed in SDS-polyacrylamide gel (10%) with gelatin (1 mg/mL) as MMPs substrate. Then, the gels were rinsed with 2.5% Triton X-100 in order to remove SDS. To activate MMPs, gels were immersed in 50 mM Tris-HCl, pH 8.0, 5 mM CaCl_2_, 5 µM ZnCl_2_, and 0.02% NaN_3_ solution and incubated overnight at 37 °C. To develop gels, Coomassie blue R-250 was used. To analyze the activity of MMPs, densitometric measurement was performed (G:BOX, Syngene, Cambridge, UK).

### 2.11. Statistical Analysis

Statistica 12 software (StatSoft, Tulsa, OK, USA) was used to perform statistical analyses of the data. A one-way ANOVA followed by Tukey’s test was applied, and the results were presented as the mean ± standard deviation (SD). Values of *p* < 0.05 were considered statistically significant.

## 3. Results

### 3.1. Interaction of VLO4 Disintegrin with Glioma Cell Lines

**Figure 1:**
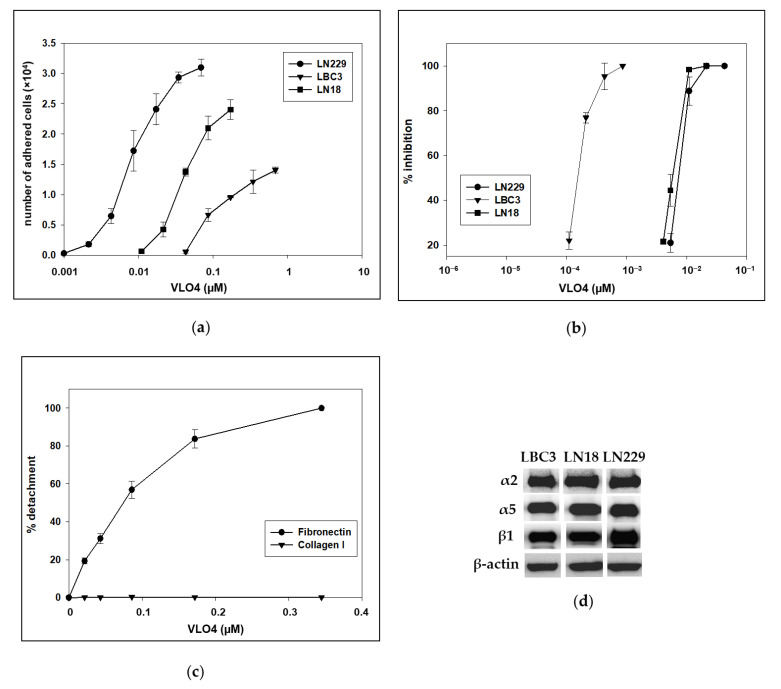
Interaction of VLO4 disintegrin with glioma cell lines. (**a**) Direct adhesion of glioma cell lines to immobilized VLO4 (number of adhered cells was calculated from a standard curve prepared from known amounts of cells on the same plate); (**b**) Inhibitory effect of VLO4 on the adhesion of glioma cell lines to immobilized fibronectin; (**c**) Effect of VLO4 on detachment of LN229 cells previously adhered to immobilized fibronectin and type I collagen. Error bars represent the mean ± SD of triplicated experiments. (**d**) Western blot showing the presence of α2β1 and α5β1 integrin in all glioma cell lines; β-actin was used as a cell protein loading control (the molecular weight markers of integrin subunits and densitometry values are shown in Figure S1).

The interaction of VLO4 with three different glioblastoma cell lines was investigated in the cell adhesion assay. Adhesion of LN18, LBC3, and LN229 cell lines to immobilized VLO4 was observed for all three cell lines, although with different potencies (Figure 1a). The most interactive cell line in this assay was the LN229 cell line, whereas the LBC3 cell line showed the lowest adhesion. The LN18 cell line was moderately active in this assay.

The next set of cell adhesion assays was devoted to testing the ability of VLO4 to inhibit cell adhesion to immobilized fibronectin, a specific ligand for α5β1 integrin. The weakest interaction with this ECM protein was observed for LBC3 cells (Figure 1b), whereas the inhibitory effect of VLO4 on the adhesion of LN18 and LN229 cells was closely similar.

In another experiment, we checked whether VLO4 binds specifically to integrin α5β1. The experiment was performed with calcein-labeled LN229 cells, which showed the most efficient binding to disintegrin immobilized on the plate, as shown in Figure 1a. The addition of VLO4 disintegrin caused cell detachment in a concentration-dependent manner only for immobilized fibronectin and not for immobilized collagen type I (Figure 1c). This activity confirms the specificity of VLO4 to interact with α5β1 integrin and not with collagen type I integrin such as a2β1, which is similar to α5β1 integrin expressed in all these cell lines (Figure 1d).

**Figure 2:**
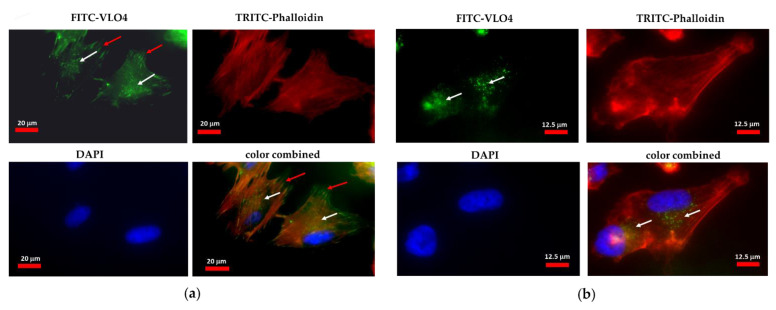
Detection of interaction of VLO4 with LN229 cells by fluorescence microscope imaging. Cells were seeded in the glass chamber and incubated with FITC-VLO4 (1 mg/mL) for 1 h at 37 °C. Cells were fixed with 4% paraformaldehyde and stained with TRITC-phalloidin (red) for cytoskeleton (F-actin) and DAPI (blue) for nuclei visualization. (**a**) Images under 400× magnification; (**b**) Images under 640× magnification. White arrows indicate areas of accumulation of granules within the cytoplasm containing internalized VLO4, and red arrows indicate accumulation near the plasma membrane.

Figure 2 shows fluorescence microscope images of LN229 cells treated with FITC-VLO4 disintegrin. The accumulation of this disintegrin was observed in the membrane areas in the form of extruded fibrils, which could be related to the interaction with focal adhesion elements. Moreover, VLO4 was internalized into the cytoplasm, which was visualized in the form of granules (possibly endosomes) near the nucleus.

### 3.2. Inhibitory Effect of VLO4 on Spreading of Glioma Cell Lines

**Figure 3:**
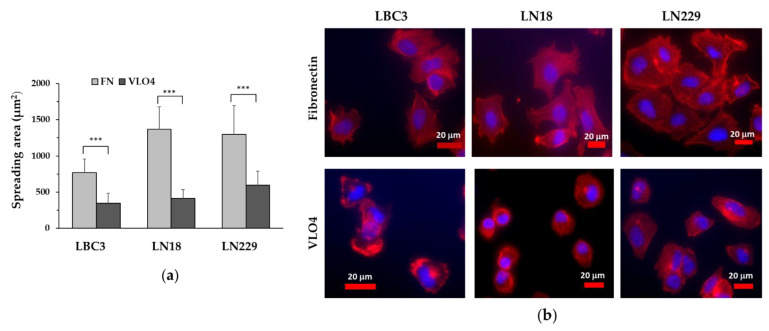
Spreading of glioblastoma cell lines on fibronectin (FN) and VLO4 disintegrin. (**a**) Graphic presentation of the spreading area per single cell. The spreading area was evaluated using MataMorph software version 7.6.5 (Molecular Devices LLC, San Jose, CA, USA). Error bars represent the mean ± SD calculated from the analysis of at least 50 cells per group; *** *p* < 0.001 indicates a statistically significant difference between cells spread on FN and VLO4. (**b**) Representative images of cells spread on the indicated ligand. F-actin, are in red, and nuclei are in blue.

The spreading of cells on the surface with immobilized fibronectin and VLO4 is shown in Figure 3. As can be seen from the graph (Figure 3a) and representative microscope images (Figure 3b), the cells of all lines spread differently; on fibronectin, the spreading is large and the cytoskeleton is apparently extended in the form of F-actin fibrils, while on VLO4, the spreading is markedly reduced and the cells are small, often spherical, and the cytoskeleton does not show the presence of F-actin fibrils.

### 3.3. Differential Effect of VLO4 on Migration of Glioma Cell Lines

**Figure 4:**
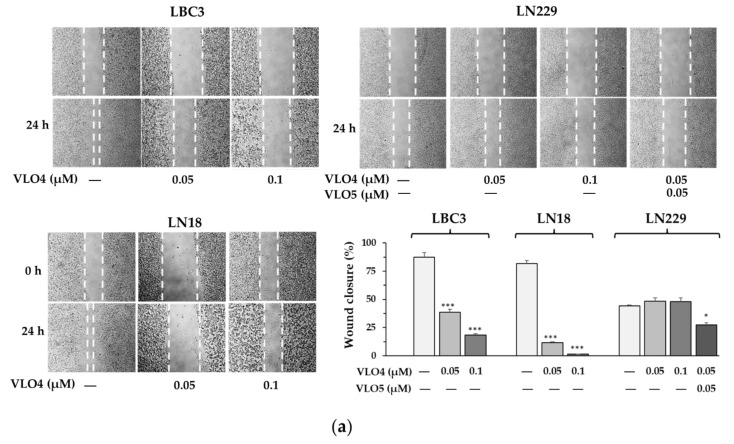

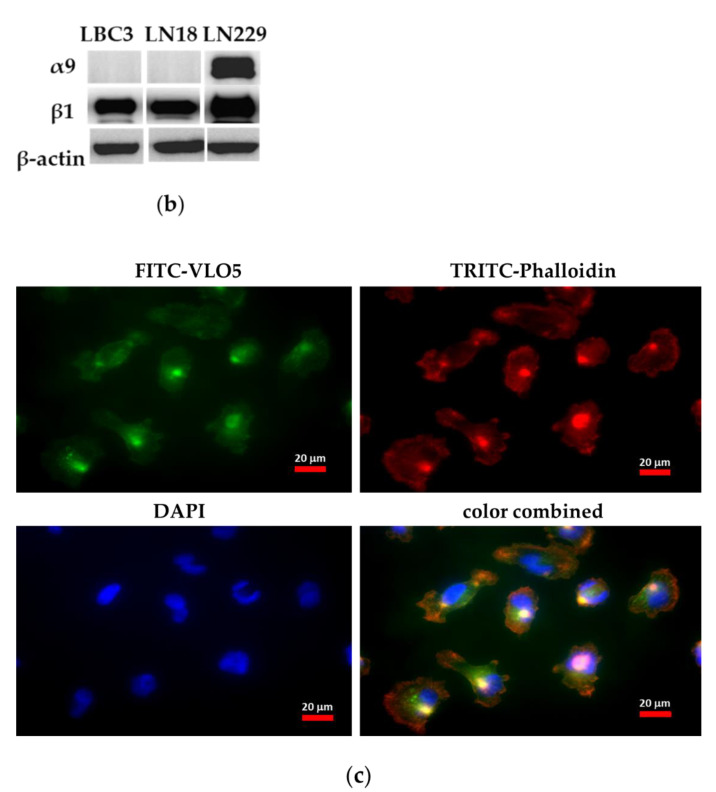
(**a**) Migration of LBC3, LN18, and LN229 cell lines after 24 h of treatment with VLO4 and VLO5 disintegrins The dashed lines mark the surface of the plate, where no cells are observed. The bars represent the results of cell migration, calculated as described in the methods, as the mean ± SD from three independent experiments: * *p* < 0.05, *** *p* < 0.001, vs. control (untreated cells). (**b**) Western blot showing the presence of α5β1 integrin in all glioma cell lines and α9β1 integrin only in LN229 cells; β-actin was used as a cell protein loading control (the molecular weight markers of integrin subunits and densitometry values are shown in Figure S4). (**c**) Detection of interaction of VLO5 with LN229 cells by fluorescence microscope imaging. Cells were seeded in a glass chamber and then incubated with FITC-VLO5 (1 mg/mL) for 1 h at 37 °C; F-actin is in red, nuclei are in blue, and VLO5 is in green.

Cell migration was investigated in the wound healing assay. VLO4 at concentrations of 0.05 and 0.1 µM after 24 h of incubation was able to significantly inhibit LBC3 and even more LN18 cell migration, whereas no effect on the LN229 cells was observed (Figure 4a). Moreover, the addition of VLO4 to the LBC3 and LN18 cell lines caused their detachment to varying degrees, most notably in LN18 cells treated with a higher concentration of VLO4 (0.1 µM). Under these conditions and at these concentrations, VLO4 did not detach LN229 cells.

The participation other than α5β1 integrin in the migration of LN229 cells was investigated by targeting α9β1 integrin, expressed only in this line (Figure 4b). This integrin is specifically antagonized by heterodimeric disintegrin, VLO5, which expresses the methionine-leucine-aspartic acid (MLD) motif in its active site. The addition of VLO5 at a concentration of 0.05 µM to cells treated with 0.05 µM VLO4 showed an inhibitory effect on LN229 cell line migration (Figure 4a). Binding of FITC-VLO5 to LN229 cells was detected by fluorescence microscope imaging (Figure 4c).

### 3.4. Decreased Expression of α5β1 Integrin in VLO4 Treated Cells

**Figure 5:**
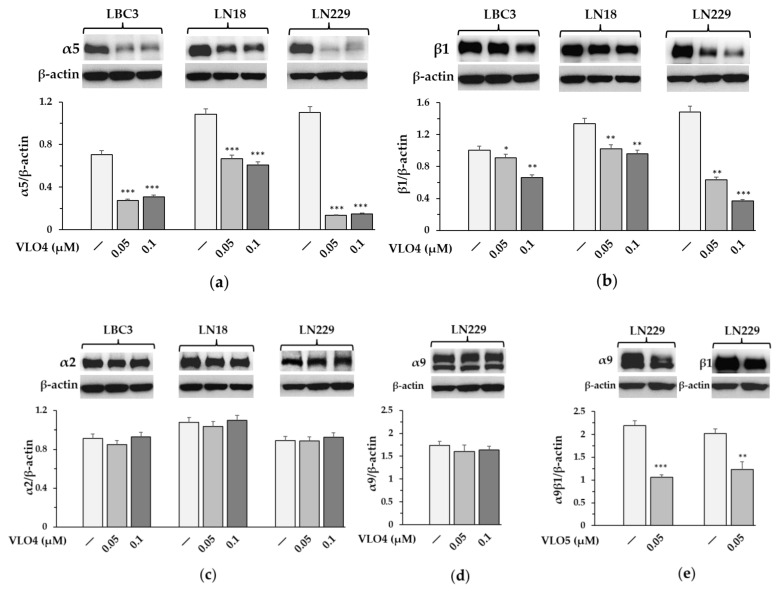
Western blot analysis of α5 (**a**), β1 (**b**), α2 (**c**), and α9 (**d**) integrin subunits in glioblastoma cell lines treated with VLO4 disintegrin as well as α9β1 integrin subunits in LN229 cell line treated with VLO5 disintegrin (**e**); β-actin was used as cell protein loading control. The bars represent the results of integrin expression as the mean ± SD from three independent experiments: * *p* < 0.05, ** *p* < 0.01, *** *p* < 0.001, vs. control (untreated cells).

Treatment of glioblastoma cells with solubilized VLO4 disintegrin resulted in a significant reduction in the expression of the α5 (Figure 5a) and β1 subunits (Figure 5b) of α5β1 integrin in all glioma cell lines. For comparison, the expression of other integrin subunits, α2 present in all cell lines (Figure 5c) and α9 present only in the LN229 line (Figure 4b and Figure 5d), was not significantly affected by the addition of this disintegrin. In turn, the expression of α9β1 integrin was downregulated by VLO5 disintegrin in this cell line (Figure 5e). The molecular weights of the determined proteins along with densitometry values are included in the Supplementary Materials (Figure S5).

### 3.5. Activity of MMP in Cells Treated with VLO4 Disintegrin

**Figure 6:**
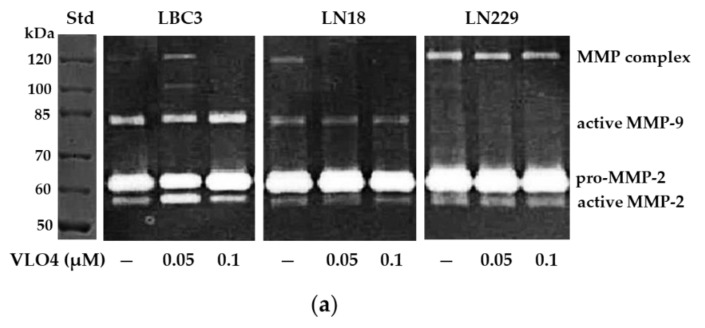

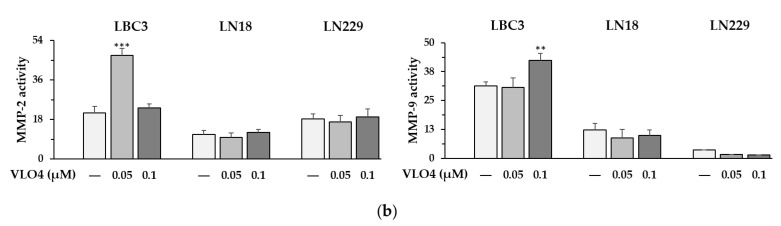
Representative gelatin zymogram (**a**). MMPs were assayed in the medium of glioblastoma cell lines treated with VLO4 disintegrin. MMPs are indicated as pro-MMP-2, active MMP-2, active MMP-9, and MMP complex (130 kDa). The molecular weight standard (Std) PageRuler Unstained Protein Ladder, ThermoFisher Scientific, Waltham, MA, USA, is shown. The bars (**b**) represent the results of MMP activity (in arbitrary units) as the mean ± SD from three independent experiments based on densitometry measurement (the values of these measurements are included in the Supplementary Materials in Figure S6); ** *p* < 0.01, *** *p* < 0.001, vs. control (untreated cells).

In all cell lines, latent and active forms of MMP-2 were detected, while active forms of MMP-9 were detected only in LBC3 and LN18. In LN229, these MMPs formed a high molecular weight complex (130 kDa) (Figure 6a). Blocking of α5β1 integrin, apart from increasing MMP-2 and MMP-9 activities in LBC3 cells treated with 0.05 and 0.1 µM VLO4, respectively, did not significantly affect the activity of these gelatinases in other cell lines (Figure 6b).

### 3.6. Differential Effect of VLO4 Disintegrin on Viability of Cells

**Figure 7:**
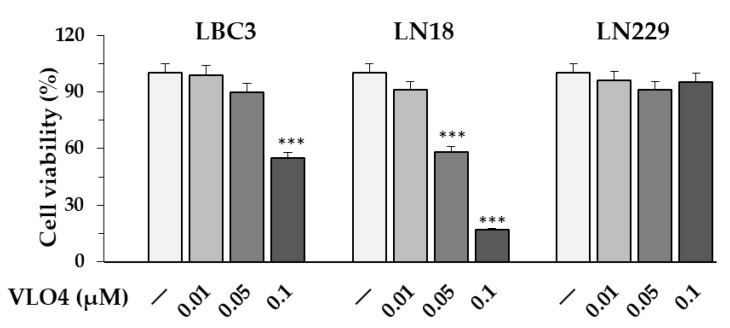
Effect of VLO4 on the viability of glioblastoma cell lines in the MTT assay. Values represent the mean ± SD of three independent experiments; *** *p* < 0.001, vs. control (untreated cells). The data are expressed as a percentage of the control sample, assumed to be 100%.

The effect of VLO4 on the cell viability of LN18, LBC3, and LN229 cell lines was assessed in an MTT assay. After 24 h of cell treatment, the inhibitory effect was only observed for LN18 and LBC3 cells and only at higher concentrations (Figure 7). The most sensitive were LN18 cells, showing reductions in cell viability of 40% and 80% under VLO4 treatment at 0.05 and 0.1 μM, respectively. LBC3 cells decrease viability only under the highest concentration of VLO4 (45% under 0.1 μM). In contrast, VLO4 at all concentrations used had no effect on LN229 cell viability. In a separate experiment, the effect of VLO5 on LN229 cells was tested. A single concentration of this disintegrin (0.05 µM) was used based on its anti-migratory effect (Figure 4a). VLO5 showed no cytotoxicity to these cells in the MTT assay under these conditions.

### 3.7. Expression of Apoptosis-Related Proteins in VLO4 Treated Cells

**Figure 8:**
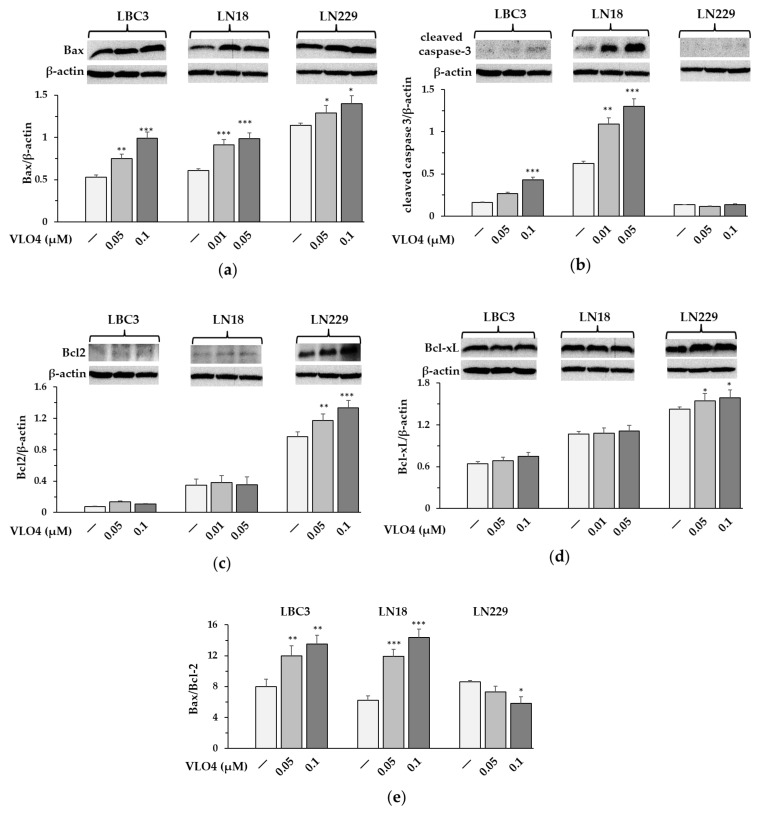
Western blot analysis of pro-apoptotic Bax (**a**) and cleaved caspase-3 (**b**) and anti-apoptotic Bcl-2 (**c**) and Bcl-xL (**d**) proteins in glioma cell lines treated with VLO4; β-actin was used as cell protein loading control. (**e**) The ratio of Bax protein to Bcl-2 protein. The bars represent the results of protein expression as the mean ± SD from three independent experiments: * *p* < 0.05, ** *p* < 0.01, *** *p* < 0.001, vs. control (untreated cells).

Western blot analysis revealed a significant increase in the expression of pro-apoptotic Bax in all cell lines treated with VLO4 (Figure 8a). Interestingly again, the most sensitive for caspase 3 activation were LN18 cells, which showed intense bands of cleaved caspase on the Western blot for both doses of VLO4 (Figure 8b). On the other hand, LBC3 cells induced cleaved caspase 3 only under the highest concentration of VLO4 and with a much lower band intensity than LN18 cells. LN229 cells were not responsive in this assay. In contrast, Bcl2 and Bcl-xL as anti-apoptotic factors remained unchanged in LBC3 and LN18, whereas they were markedly increased in LN229 in the presence of both concentrations of VLO4 (Figure 8c,d). The ratio of Bax protein to Bcl-2 protein increased in a concentration-dependent manner in VLO4-treated LBC3 and LN18 cell lines but decreased in the LN229 cell line (Figure 8e). The molecular weights of the determined proteins along with densitometry values are included in the Supplementary Materials (Figure S8).

## 4. Discussion

Glioblastoma (CNS WHO grade 4) is still the most difficult tumor to treat [6,7,8,9,12,13,14]. Regardless of the many different treatment strategies, new treatment methods are urgently needed due to the dismal prognosis of GB patients.

Integrins as cell surface receptors may be an interesting target to repress the development of this pathology, at least as a support for chemo/radiotherapy and surgical intervention. In this study, we focused on blocking α5β1 integrin using RGD homodimeric snake venom disintegrin, VLO4. Using the adhesion assay, we demonstrated the selective binding of VLO4 to α5β1 integrin in three GBM cell lines: LBC3, LN18, and LN229. All these lines interact with VLO4, albeit with different potencies. This interaction was shown in the direct adhesion of the immobilized disintegrin and in competition with the adhesion of the immobilized natural ligand of α5β1 integrin, fibronectin. The results revealed that the LBC3 cell line adheres to the immobilized VLO4 to the lowest extent, and soluble disintegrin is the most effective way to block adhesion of this cell line to immobilized fibronectin. Two other cell lines showed closely similar responses for blocking adhesion to fibronectin, although in direct adhesion to immobilized VLO4, the LN229 cells were more effective. Therefore, the LN229 cell line was used to test the detachment of already adhered cells from the fibronectin matrix. VLO4 appeared to be a potent detachment factor for this cell line, despite its strongest binding to this ECM protein.

The differential interaction of VLO4 with these three cell lines could be explained by the different expression levels of α5β1 integrin on their surfaces. Indeed, Western blots displayed a lower expression of this integrin in LBC3 cells but were similar in LN18 and LN229 cells. However, expression level cannot completely explain such a big difference in activity among these cell lines. It is likely that activation of α5β1 integrin occurs differently in these cells. It may be associated with the presence of other supporting molecules (co-receptors) present on the plasma membrane. Previously, we found the association of α9β1 integrin with the common neurotrophin receptor, p75^NTR^ [38]. Additional work is required to find out if any plasma membrane protein is associated with α5β1 integrin to enhance its pro-adhesive properties.

The wound healing assay revealed that VLO4 inhibited cell migration in LBC3 and LN18 cell lines. However, this disintegrin had no effect on the LN229 cell line in this assay. This event may be explained by the presence of other integrins on the LN229 cell surface, which promote cell migration and reduce or completely diminish the effect of α5β1 integrin. The best candidate was α9β1 integrin, which is expressed on the LN229 cells but not on the two other cell lines investigated in this assay. Therefore, we used a specific snake venom disintegrin, VLO5, to test it in a migration assay. Indeed, VLO5 resulted in the reduced migration of LN229 cells; however, in combination with VLO4. These results clearly indicate that the pro-invasive properties of glioma cells depend on the presence of these two integrins. Previously, we considered α9β1 integrin as a potential therapeutic target for the therapy of glioma [26]. Expression of this receptor has been found only on cells present in GBM tissue and absent in normal human brain tissue [26]. This integrin contributed to malignancy, and its overexpression in glioma tissue increased the diffusive properties, proliferation, and survival of cancer cells [26]. It turned out to be interesting that this integrin, unlike most other integrins, can regulate specific mechanisms of cell migration. α9β1, utilizing the spermidine/spermine-N1-acetyltransferase (SSAT)-inward rectifier potassium channel (Kir) pathways along with the common integrin signaling pathway (Src, FAK), is able to accelerate cell migration, while SSAT knockdown reduced the migration of glioma xenograft cells [27]. These mechanisms may be responsible for the greater resistance of the LN229 line (α9β1-positive) to VLO4 treatment compared to the α9β1-negative lines (LBC-3 and LN18), which was observed in our study; only the addition of VLO5 targeting α9β1 weakened cell migration. Overexpression of sodium-calcium exchanger (NCX) was demonstrated in both GBM cell lines and biopsies from GBM patients, while NCX blockade impaired lamellipodia formation and GBM cell migration [40,41]. These results confirm the very important role of ion channels and exchangers in the migration and invasiveness of GBM.

There is also evidence that integrins, including α5β1, can induce the expression and modulate the action of different proteases, especially MMP-2 and MMP-9 [42,43,44]. VLO4 as an antagonist of α5β1 integrin has not inhibited any activity of these enzymes, but in reverse, it activated MMP-2 and MMP-9 in LBC3 cells. This agonistic effect on this cell line is difficult to explain. It may be correlated with different membrane environments, which induce unusual signals for LN18 and LN229 cell lines. GBM is characterized by an unstable pathology, making this tumor difficult to treat. For example, the expression of MMP-9 in the LN229 line is different than in two other cell lines. The active form of MMP-9 was not found in this cell line, but instead strong expression of the MMPs complex was detected. These results indicate that using snake venom disintegrins may have a variable effect on MMPs-dependent mechanisms of invasion of gliomas.

Integrins have been characterized as receptors that, under certain circumstances, may induce pro-apoptotic cell stimuli. It may happen following their binding to some ligands (e.g., degraded ECM) or by cell detachment (anoikis) [45,46]. In the presented work, we found that soluble VLO4 decreased cell survival ratio, as demonstrated by the cell viability assay, MTT. However, this effect was only noted for LBC3 and LN18 but not for the LN229 cell line. Further, we tested the association of this effect with the induction of apoptosis, and we found a correlation. Expression of the pro-apoptotic factor Bax was increased in LBC3 and LN18 cell lines to a greater extent than in LN229 cells. Activation of executor caspase 3 was observed also for LBC3 and LN18 cells, but not for LN229. Moreover, expression of mitochondrial anti-apoptotic Bcl2 (B cell lymphoma/leukemia 2) and anti-apoptotic Bcl-xL was upregulated in LN229 cells and not changed in two other cell lines. The calculated ratio of Bax/Bcl2 showed the pro-apoptotic effect of VLO4 in LBC3 and LN18 cells, whereas in LN229 cells it rather prevailed as a preventive measure against apoptosis activity. These data suggest that snake venom disintegrins may induce different death/survival effects in different cells. The pro-apoptotic activity of VLO4 on LBC3 and LN18 cell lines is in agreement with that of another RGD dimeric disintegrin, lebein, which induces apoptosis in colon cancer cells [47]. On the other hand, MLD dimeric disintegrin, VLO5, was found to be an α9β1 integrin-dependent inducer of apoptosis in LN229 cells [26], whereas it reduced spontaneous apoptosis in neutrophils isolated from human blood [48].

One of the possible mechanisms of the antagonistic effect of disintegrins against integrins is a reduction in the expression of these receptors. The results presented in this paper revealed that treatment of all three glioma cell lines with VLO4 in its soluble form decreased the expression of both subunits α5 and β1. In contrast, the expression of a2 and a9 subunits, which are not involved in the creation of receptors for VLO4, was not affected by this disintegrin. In addition, we tested in this assay another disintegrin, VLO5, which is a ligand for α9β1 integrin. This integrin is only expressed in the LN229 cell line, where VLO5 reduces its expression. Therefore, we can generally conclude that disintegrins are the factors that affect the presence of integrins in the cells. The mechanism of action of disintegrins in this cellular outcome may be related to the endosomal internalization of the integrin/disintegrin complex and lysosomal degradation. The fluorescence imaging using FITC-labeled disintegrins revealed the presence of disintegrins in their granular forms in the cytoplasm. Further studies will explain the possibility of disintegrin-dependent lysosomal degradation of integrins. Interestingly, immobilized VLO4 in its solid form has not shown any toxic effect (no signs of pro-apoptotic effect). However, the spreading of glioma cells on the VLO4 matrix was very limited if compared with the natural ligand, fibronectin. It should be noted that the cytoskeletal actin filaments were not completely developed. These results suggest that VLO4 may be used as a pro-adhesive matrix for selective delivery of chemotherapeutics to cancer cells in the form of, e.g., nanoparticles.

Disintegrins isolated from the venom of various snake species show therapeutic potential in many other cancers. Leberagin-C, derived from the venom of *Macrovipera lebetina transmediterrannea*, antagonizes α5β1, αvβ3, and αvβ6 integrins and inhibits adhesion, migration, and invasion of breast cancer cells (MDA-MB-231 and D3H2LN) [49]. In addition, in vivo experiments revealed a 50% reduction in tumor angiogenesis and a 73% reduction in D3H2LN xenograft tumor size after 21 days of twice-weekly treatment with a concentration of 2 µM [49]. Previous research by these authors showed the ability of this disintegrin to prevent melanoma cancer cells from adhering to fibrinogen and fibronectin [50]. Another disintegrin, Contorstatin, isolated from the venom of the southern copperhead snake (*Agkistrodon contortrix contortrix*), also has potential in melanoma therapy. It binds to αvβ3 integrin but also recognizes other integrins, including α5β, and showed an inhibiting effect on tumor growth, neovascularization, and metastases [51]. Recombinant disintegrin (Vicrostatin), obtained by fusing the N-terminal amino acids from Contrastatin and the C-terminal amino acids from echistatin, is among the most advanced in preclinical studies. It targets many integrins, including α5β, and exerts potent antitumor and antiangiogenic effects in a wide variety of preclinical animal models of cancer (breast, prostate, ovarian, and glioblastoma) [51]. ^131^IVitronectin combined with TMZ therapy resulted in slightly better treatment results than radiation combined with TMZ in GBM mice [52]. Lebein, an heterodimeric disintegrin isolated from *Macrovipera lebetina* snake venom, inhibited growth and angiogenesis rates of tumor xenografts in colon cancer cells, which was accompanied by significantly reduced expression of α5β1 integrin [47], similarly to VLO4-treated glioma cell lines in our study. All these studies indicate the enormous potential of these molecules in cancer therapy. However, there are limitations to the use of peptide-based drugs, which include, among others, instability, immunogenicity, and availability [36]. Instability and immunogenicity can be reduced by liposomal delivery of disintegrins, which increases the half-life in circulation and passive accumulation in the tumor, and in this form they do not show platelet reactivity and are not recognized by the immune system [53,54]. However, the use of disintegrins is more beneficial compared to the use of antibodies in cancer therapy. They have a shorter half-life, are easier to control, are susceptible to inhibition, show lower immunogenicity, have greater availability, and are less expensive compared to antibodies [36].

## 5. Conclusions

Our results provide new data on the antitumor effects of VLO4 disintegrin, which may have implications for the development of integrin-targeted therapy for glioblastoma. The activity of VLO4 is associated with its interaction with α5β1 integrin, which is expressed on all tested cell lines. The lower effect of this disintegrin on the pro-invasive properties of the LN229 cell line may be related to the expression of α9β1 integrin; theref ore, the combination of antagonists of both integrins, VLO4 (α5β1) and VLO5 (α9β1), may be more effective for the invasion of glioma cells in non-affected tumor areas of the brain.

In the presented article, we summarized the initial studies, which will be continued in the future. Additional experiments are required to increase the number of glioma cell lines and possibly primary cells isolated from the patients’ tissue, especially those expressing α9β1 integrin. The next approach will be in vivo experiments in the glioma animal model, such as intracranial implantation of human cell lines in nude rodents. Application of VLO4 in these models will answer the general question about the effectiveness of blocking α5β1 integrin in the development of this tumor.

## Data Availability

Data available on request.

## Supplementary Materials

The following supporting information can be downloaded at: https://www.mdpi.com/article/10.3390/cancers15194805/s1, Figure S1: For Figure 5a, Western blot of α5 integrin (A) and β-actin (B) in glioblastoma cell lines LBC3, LN18 and LN229 treated with VLO4 disintegrin. The membranes were cut at a height between the 63 and 75 kDa markers and the upper part of the membrane was used to identify integrin α5 and the lower part for β-actin, which allowed to study the expression of tested and reference proteins under the same conditions and in the same samples. Densitometry values of the protein bands and the molecular weight markers are also shown; Figure S2: For Figure 5b, Western blot of integrin β1 (A) and β-actin (B) in glioblastoma cell lines LBC3, LN18 and LN229 treated with VLO4 disintegrin. The membranes were cut at a height between the 63 and 75 kDa markers and the upper part of the membrane was used to identify integrin β1 and the lower part for β-actin or another low-molecular tested protein, which allowed to study the expression of tested and reference proteins under the same conditions and in the same samples. Densitometry values of the protein bands and the molecular weight markers are also shown; Figure S3: For Figure 5c, Western blot of integrin α2 (A) and β-actin (B) in glioblastoma cell lines LBC3, LN18 and LN229 treated with VLO4 disintegrin. The membranes were cut at a height between the 63 and 75 kDa markers and the upper part of the membrane was used to identify integrin α2 and the lower part for β-actin or another low-molecular tested protein, which allowed to study the expression of tested and reference proteins under the same conditions and in the same samples. Densitometry values of the protein bands and the molecular weight markers are also shown. Figure S4: For Figure 5d,e, Western blot of α9β1 integrin (A) and β-actin (B) in glioblastoma cell line LN229 treated with VLO4 and VLO5 disintegrins. The membranes were cut at a height between the 63 and 75 kDa markers and the upper part of the membrane was used to identify integrin α9 and β-1 subunits and the lower part for β-actin or another low-molecular protein, which allowed to study the expression of tested and reference proteins under the same conditions and in the same samples. Densitometry values of the protein bands and the molecular weight markers are also shown. Figure S5: For Figure 8a, Western blot of Bax (A) and β-actin (B) in glioblastoma cell lines LBC3, LN18 and LN229 treated with VLO4 disintegrin. Densitometry values of the protein bands and the molecular weight markers are also shown. Figure S6: For Figure 8b, Western blot of cleaved caspase-3 (A) and β-actin (B) in glioblastoma cell lines LBC3, LN18 and LN229 treated with VLO4 disintegrin. The membranes were cut at a height between the 25 and 48 kDa markers and the lower part of the membrane was used to identify cleaved caspase 3 and the upper part for high-molecular tested proteins. Densitometry values of the protein bands and the molecular weight markers are also shown. Figure S7: For Figure 8c, Western blot of Bcl2 (A) and β-actin (B) in glioblastoma cell lines LBC3, LN18 and LN229 treated with VLO4 disintegrin. The membranes were cut at a height between the 48 and 75 kDa markers and the lower part of the membrane was used to identify Bcl2 and the upper part for another tested high-molecular protein. Densitometry values of the protein bands and the molecular weight markers are also shown. Figure S8: For Figure 8d, Western blot of Bcl-xL (A) and β-actin (B) in glioblastoma cell lines LBC3, LN18 and LN229 treated with VLO4 disintegrin. Densitometry values of the protein bands and the molecular weight markers are shown. Figure S9: For Figure 6, Gelatin zymography. MMPs were assayed in the medium of glioblastoma cell lines LBC3, LN18 and LN229 treated with VLO4 disintegrin. MMPs are indicated as pro-MMP-2, active MMP-2, active MMP-9 and MMP complexes (130 and 240 kDa). The molecular weight standard (kDa) and densitometry values are shown. Figure S10: For Figure 1d and Figure 4b, Western blot of integrin subunits. The molecular weight markers and densitometry values are shown.
