# Supplementary material for: Effect of Dimeric Disintegrins Isolated from Vipera lebetina obtusa Venom on Glioblastoma Cellular Responses
Source: Cancers (Basel). 2023 Sep 29;15(19):4805. doi: 10.3390/cancers15194805 (PMC10572073; doi:10.3390/cancers15194805)

Supplemental Materials

Article

Effect of Dimeric Disintegrins Isolated from *Vipera lebetina obtusa* Venom on Glioblastoma Cellular Responses

Anna Galicka [1]*, Łukasz Szoka [2], Iwona Radziejewska [1], Cezary Marcinkiewicz [3]*

[1] Department of Medical Chemistry, Medical University of Bialystok, Mickiewicza 2A, 15-222 Bialystok, Poland; iwona@umb.edu.pl
[2] Department of Medicinal Chemistry, Medical University of Bialystok, Mickiewicza 2D, 15-222 Bialystok, Poland; lukasz.szoka@umb.edu.pl
[3] Department of Bioengineering, Temple University CoE, Philadelphia, PA 19406, USA
* Correspondence: angajko@umb.edu.pl (A.G.); cezary.marcinkiewicz@temple.edu (C.M.)

Figure S1. For Figure 5a, Western blot of α5 integrin (A) and β-actin (B) in glioblastoma cell lines LBC3, LN18 and LN229 treated with VLO4 disintegrin. The membranes were cut at a height between the 63 and 75 kDa markers and the upper part of the membrane was used to identify integrin α5 and the lower part for β-actin, which allowed to study the expression of tested and reference proteins under the same conditions and in the same samples. Densitometry values of the protein bands are also shown.lar weight markers

(A)

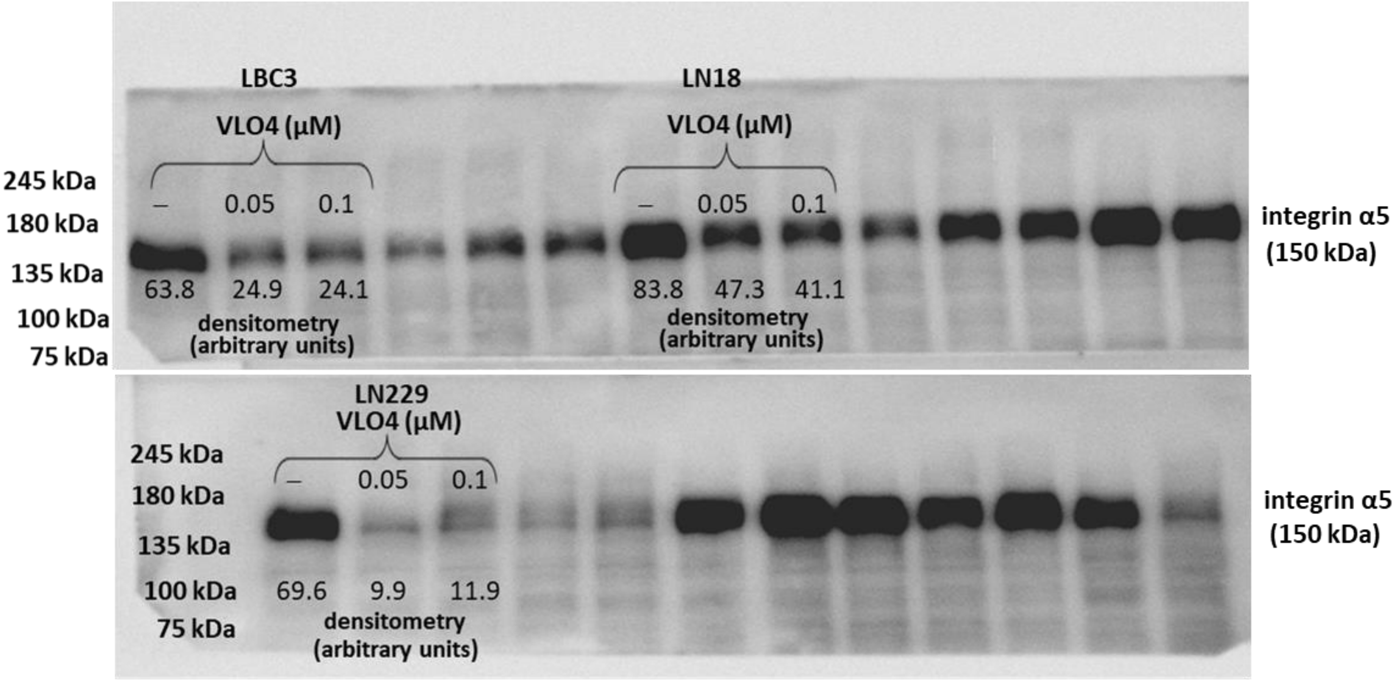

molecular weight marker

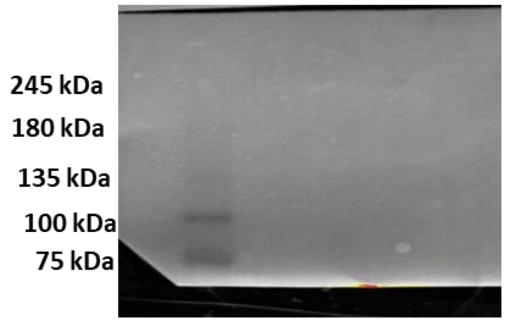

(B)

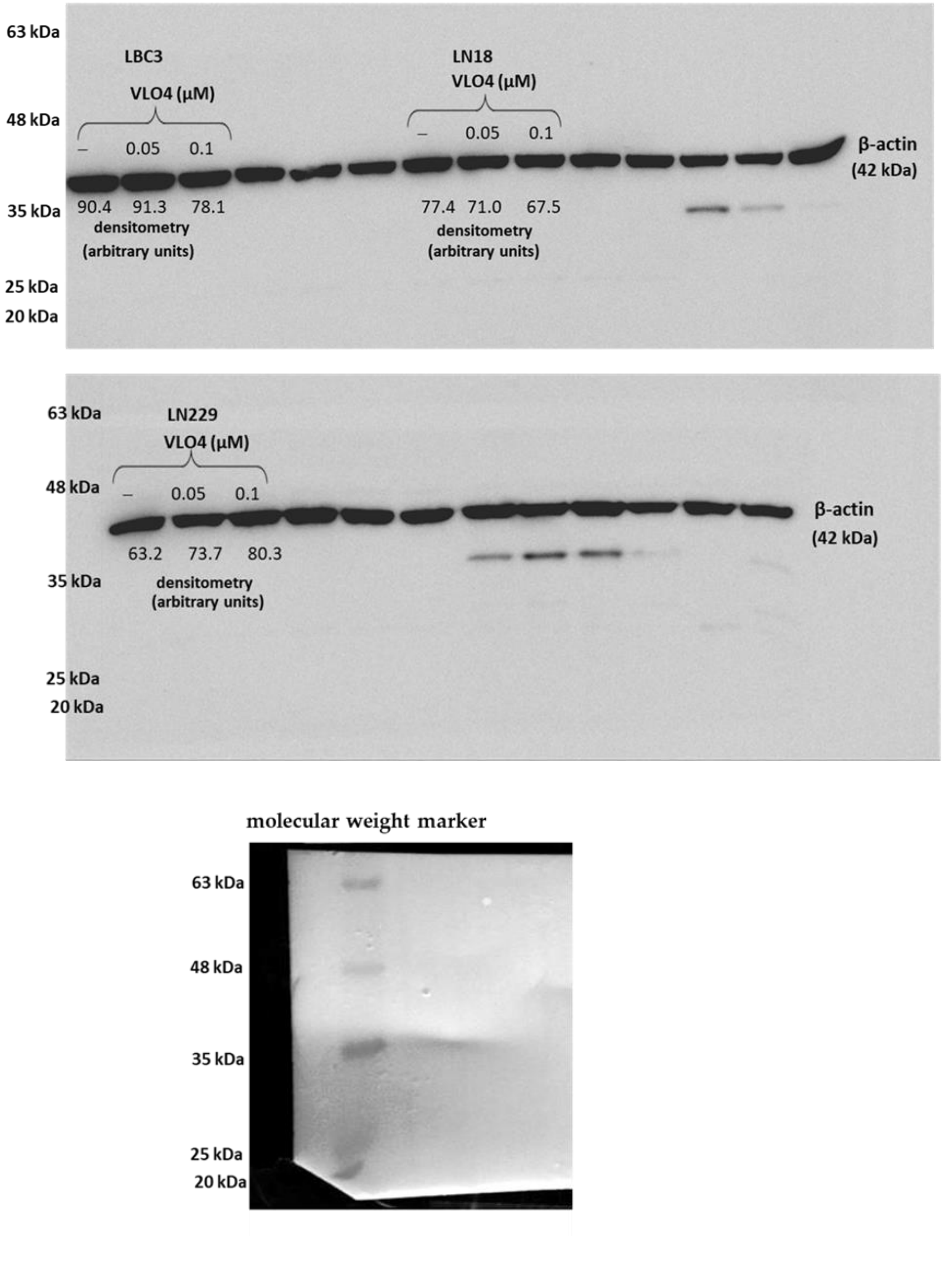
63 kDa
48 kDa
35 kDa
25 kDa
20 kDa
LBC3
VLO4 (μM)
–
0.05
0.1
90.4
91.3
78.1
densitometry
(arbitrary units)
LN18
VLO4 (μM)
–
0.05
0.1
77.4
71.0
67.5
densitometry
(arbitrary units)
β-actin
(42 kDa)
63 kDa
48 kDa
35 kDa
25 kDa
20 kDa
LN229
VLO4 (μM)
–
0.05
0.1
63.2
73.7
80.3
densitometry
(arbitrary units)
β-actin
(42 kDa)
molecular weight marker
63 kDa
48 kDa
35 kDa
25 kDa
20 kDa

Figure S2. For Figure 5b, **Western blot of integrin β1 (A) and β-actin (B) in glioblastoma cell lines LBC3, LN18 and LN229 treated with VLO4 disintegrin.** The membranes were cut at a height between the 63 and 75 kDa markers and the upper part of the membrane was used to identify integrin β1 and the lower part for β-actin or another low-molecular tested protein, which allowed to study the expression of tested and reference proteins under the same conditions and in the same samples. Densitometry values of the protein bands and the molecular weight markers are also shown.

(A)

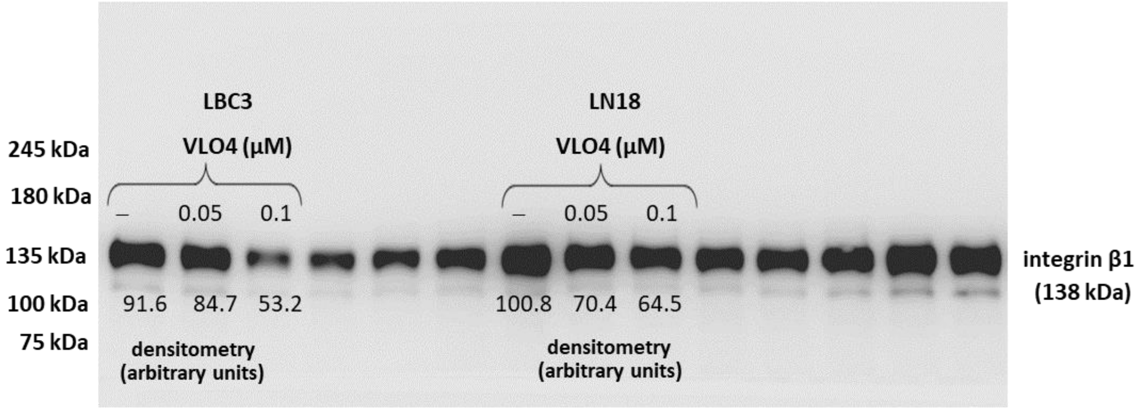

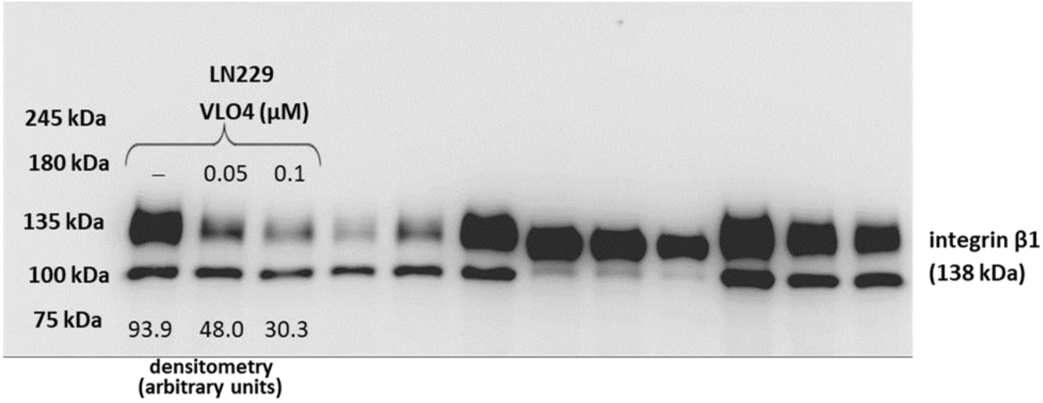

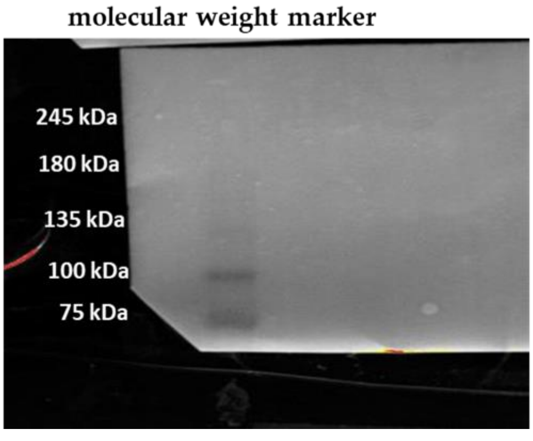

(B)

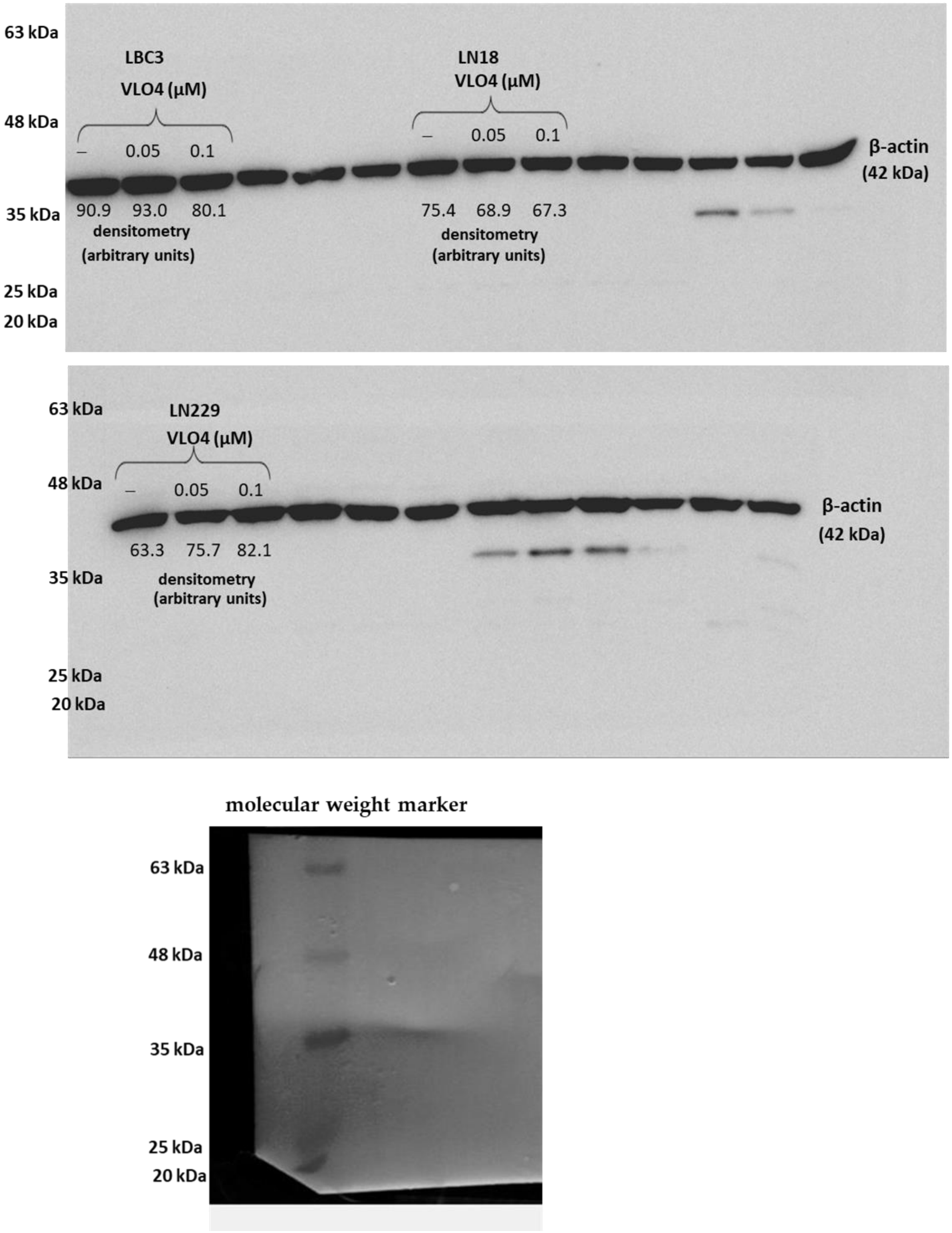
63 kDa
48 kDa
35 kDa
25 kDa
20 kDa
LBC3
VLO4 (µM)
–
0.05
0.1
90.9
93.0
80.1
densitometry
(arbitrary units)
LN18
VLO4 (µM)
–
0.05
0.1
75.4
68.9
67.3
densitometry
(arbitrary units)
β-actin
(42 kDa)
63 kDa
48 kDa
35 kDa
25 kDa
20 kDa
LN229
VLO4 (µM)
–
0.05
0.1
63.3
75.7
82.1
densitometry
(arbitrary units)
β-actin
(42 kDa)
molecular weight marker
63 kDa
48 kDa
35 kDa
25 kDa
20 kDa

Figure S3. For Figure 5c, Western blot of integrin $\alpha 2$ (A) and β-actin (B) in glioblastoma cell lines LBC3, LN18 and LN229 treated with VLO4 disintegrin. The membranes were cut at a height between the 63 and 75 kDa markers and the upper part of the membrane was used to identify integrin $\alpha 2$ and the lower part for β-actin or another low-molecular tested protein, which allowed to study the expression of tested and reference proteins under the same conditions and in the same samples. Densitometry values of the protein bands and the molecular weight markers are also shown.

(A)

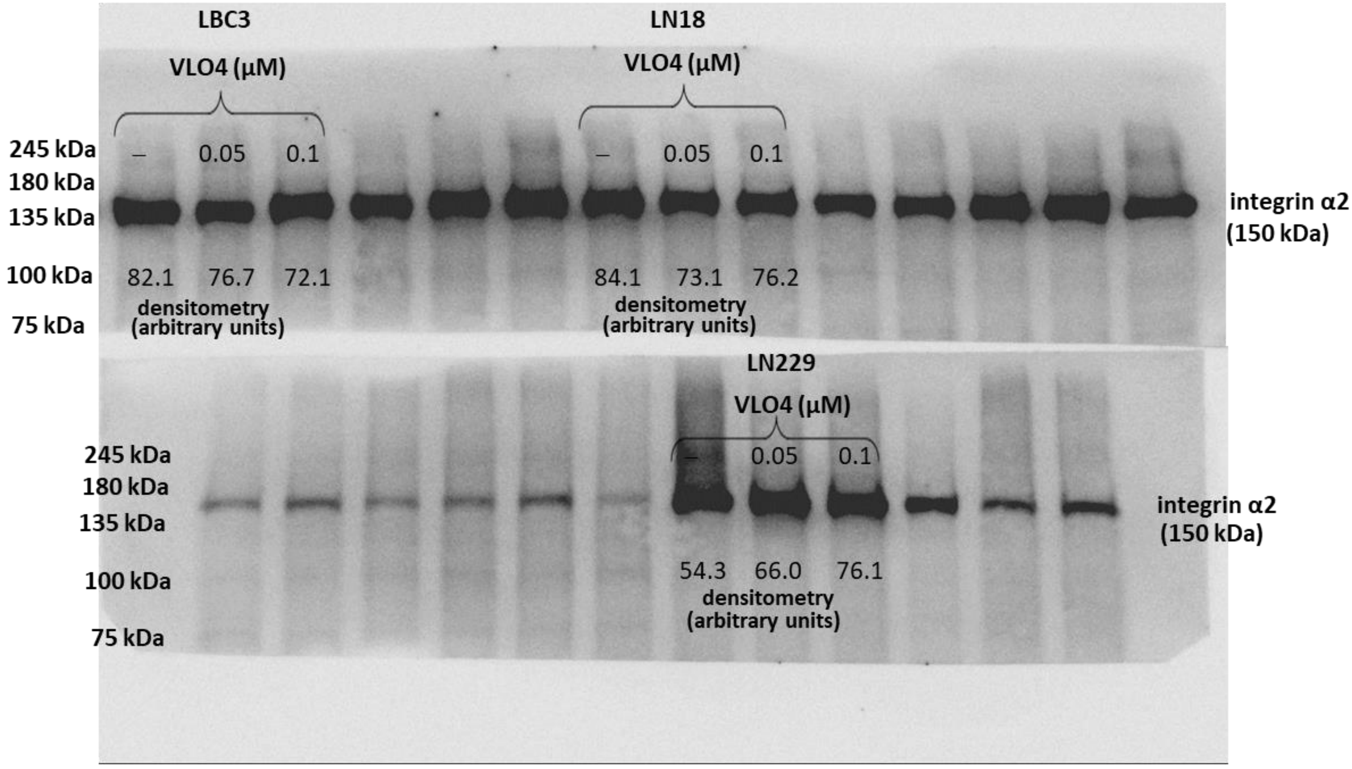

molecular weight marker

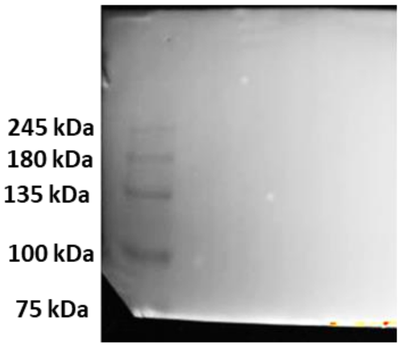

(B)

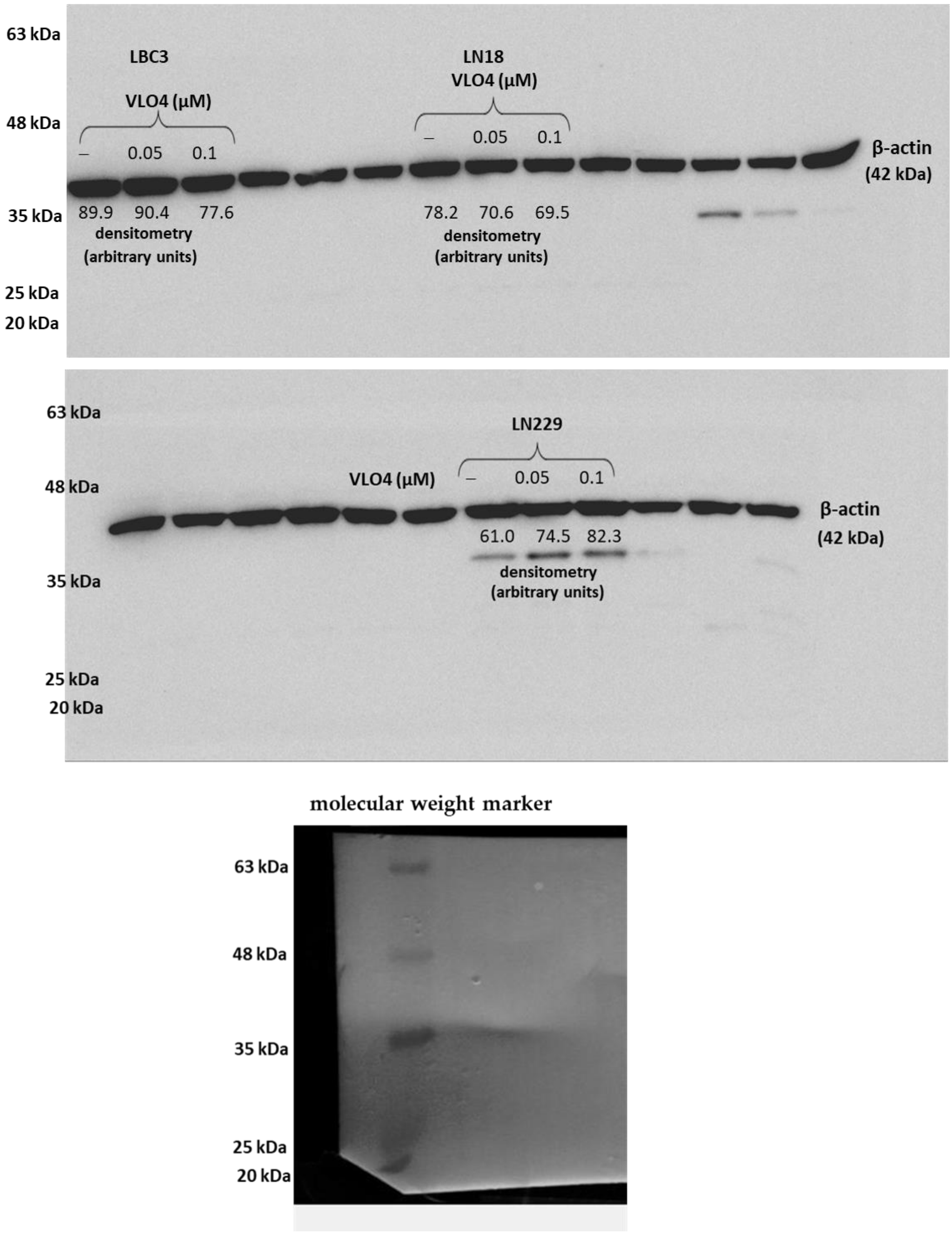

63 kDa
48 kDa
35 kDa
25 kDa
20 kDa
LBC3
VLO4 (μM)
– 0.05 0.1
89.9 90.4 77.6
densitometry (arbitrary units)
LN18
VLO4 (μM)
– 0.05 0.1
78.2 70.6 69.5
densitometry (arbitrary units)
β-actin (42 kDa)
63 kDa
48 kDa
35 kDa
25 kDa
20 kDa
LN229
VLO4 (μM)
– 0.05 0.1
61.0 74.5 82.3
densitometry (arbitrary units)
β-actin (42 kDa)
molecular weight marker
63 kDa
48 kDa
35 kDa
25 kDa
20 kDa

Figures S4. For Figure 5d,e, Western blot of $\alpha 9\beta 1$ integrin (A) and β-actin (B) in glioblastoma cell line LN229 treated with VLO4 and VLO5 disintegrins. The membranes were cut at a height between the 63 and 75 kDa markers and the upper part of the membrane was used to identify integrin $\alpha 9$ and β-1 subunits and the lower part for β-actin or another low-molecular protein, which allowed to study the expression of tested and reference proteins under the same conditions and in the same samples. Densitometry values of the protein bands and the molecular weight are also shown.

(A)

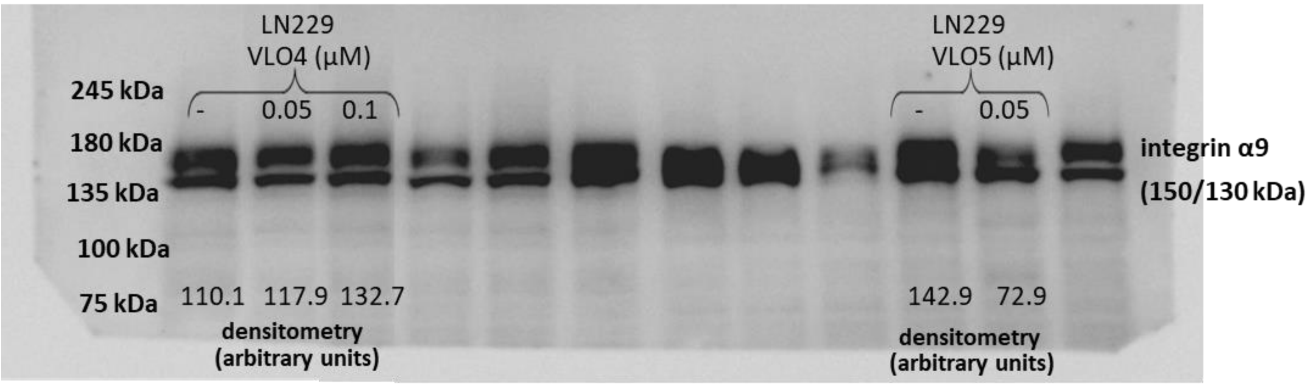

molecular weight marker

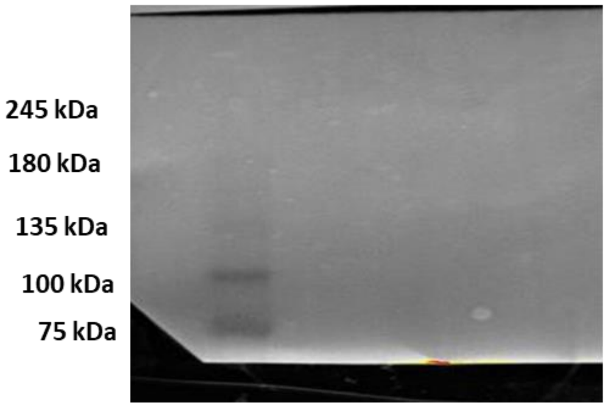

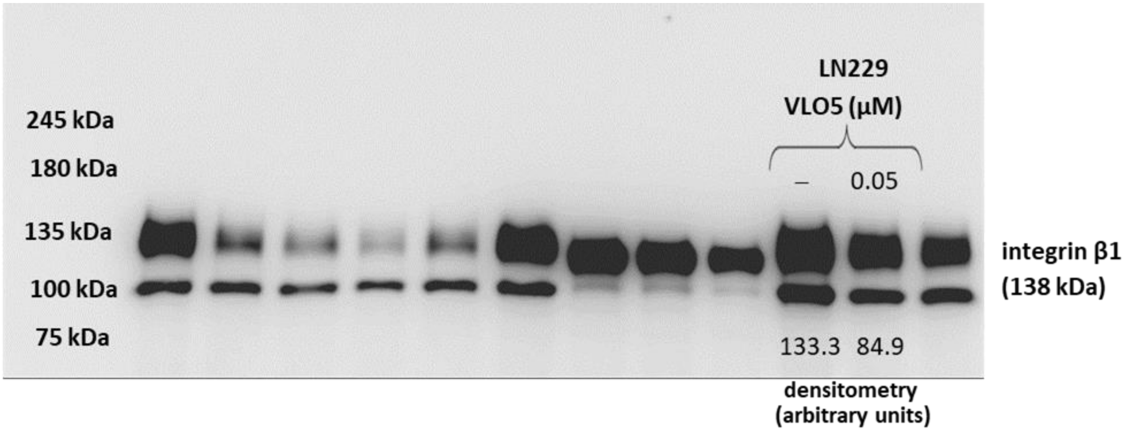

molecular weight marker

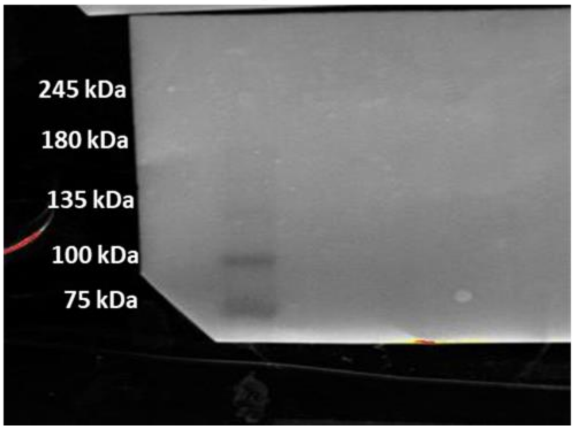

(B)

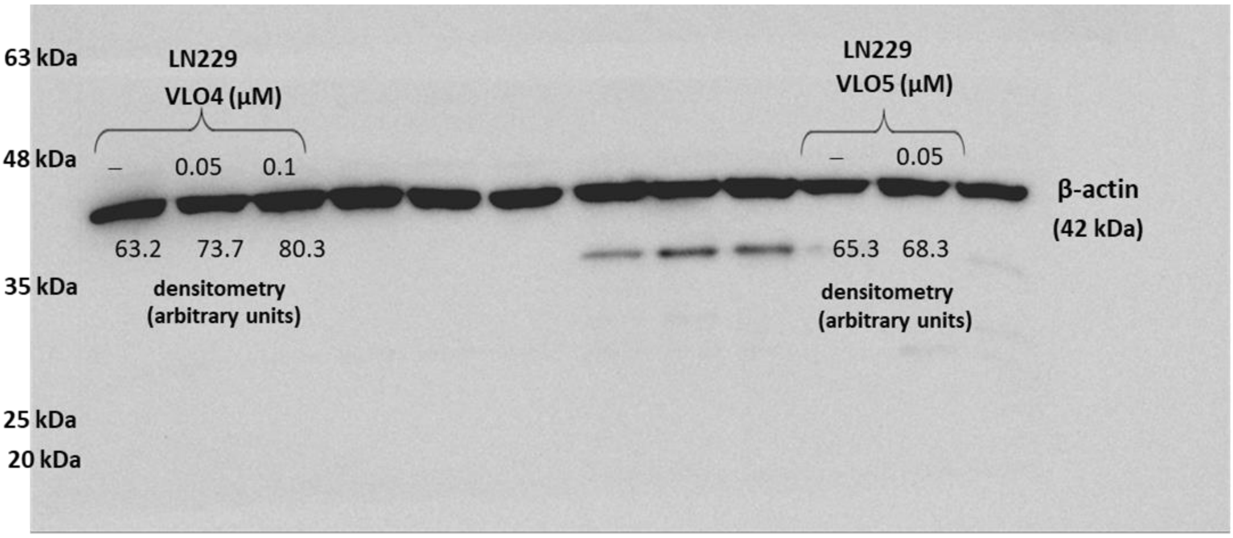

molecular weight marker

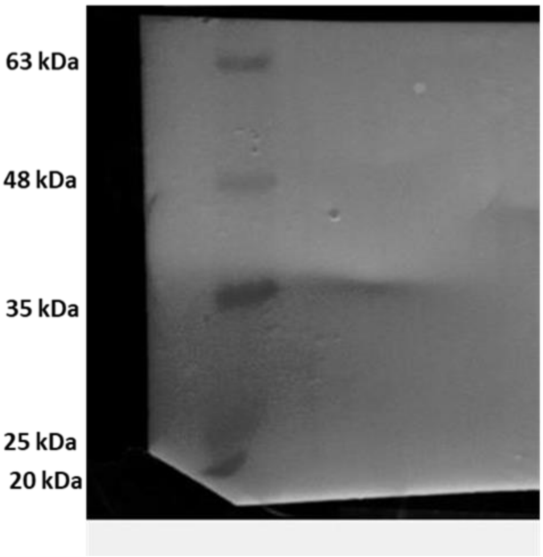

Figure S5. For Figure 8a, Western blot of Bax (A) and β-actin (B) in glioblastoma cell lines LBC3, LN18 and LN229 treated with VLO4 disintegrin. Densitometry values of the protein bands and the molecular weight markers are also shown.

(A)

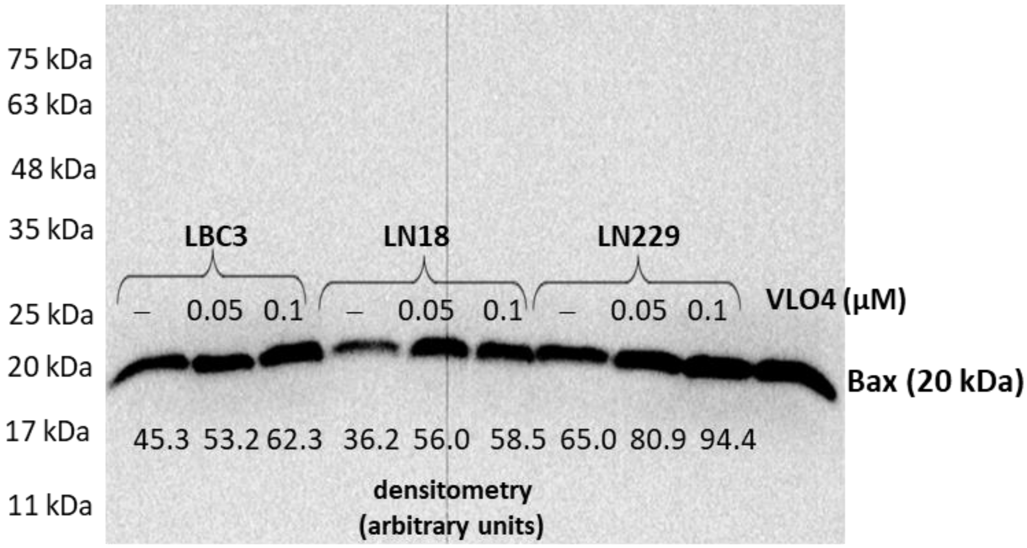

molecular weight marker

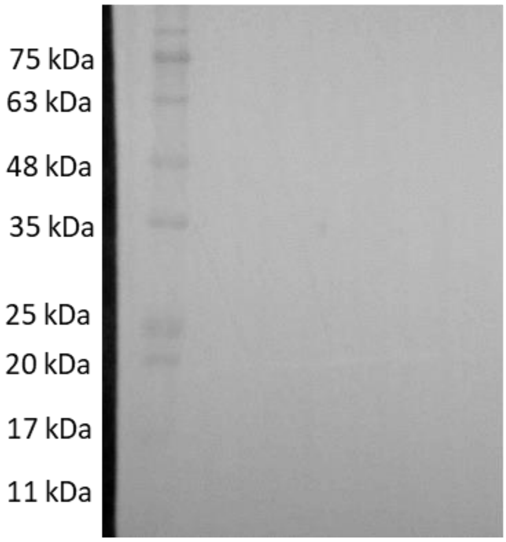

(B)

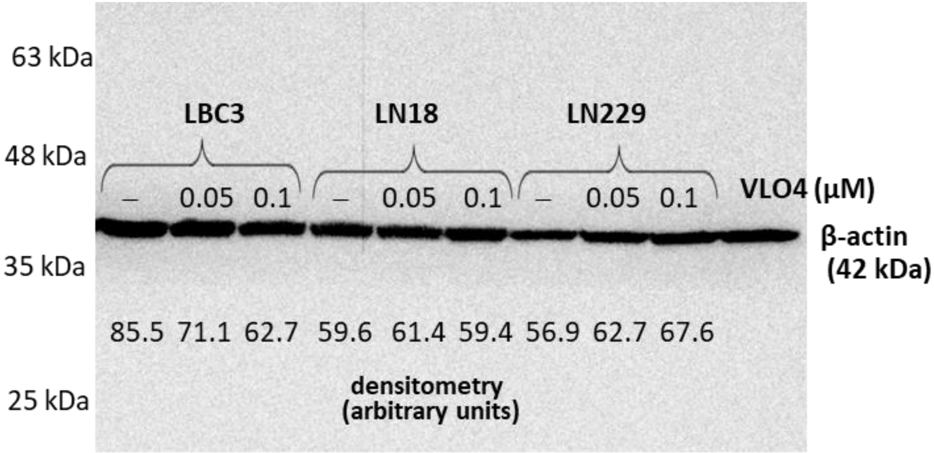

molecular weight marker

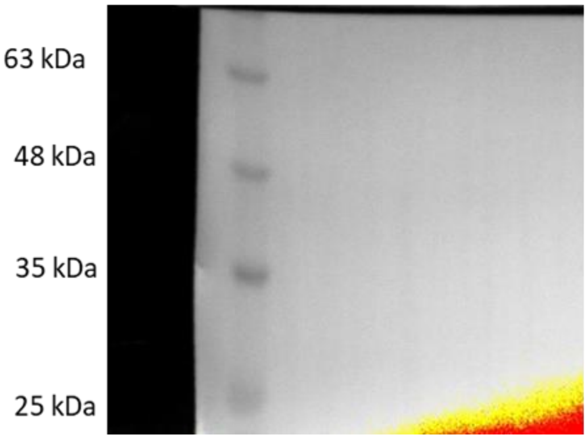

Figure S6. For Figure 8b, Western blot of cleaved caspase-3 (A) and β-actin (B) in glioblastoma cell lines LBC3, LN18 and LN229 treated with VLO4 disintegrin. The membranes were cut at a height between the 25 and 48 kDa markers and the lower part of the membrane was used to identify cleaved caspase 3 and the upper part for high-molecular tested proteins. Densitometry values of the protein bands and the weight markers are also shown.

(A)

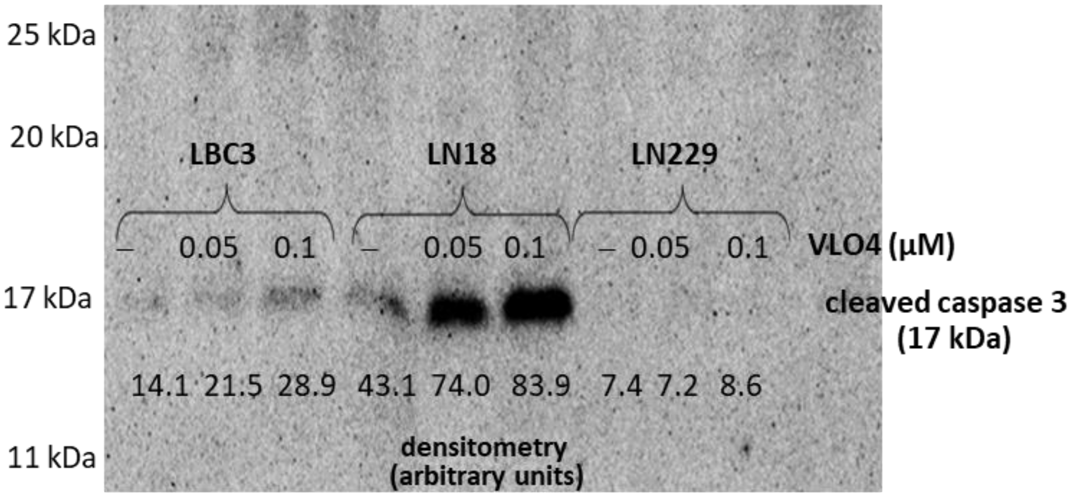

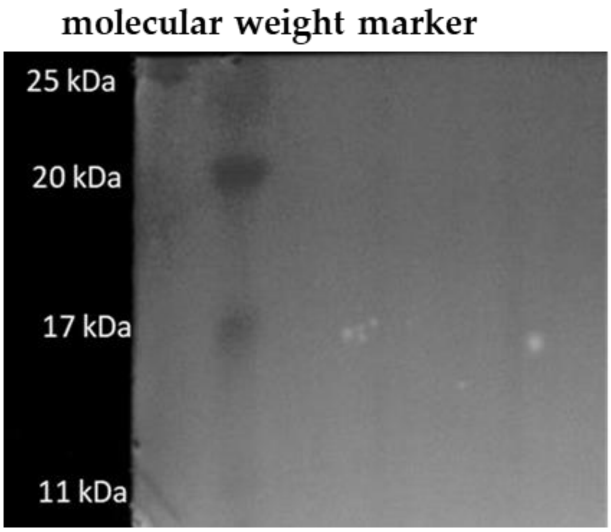

(B)

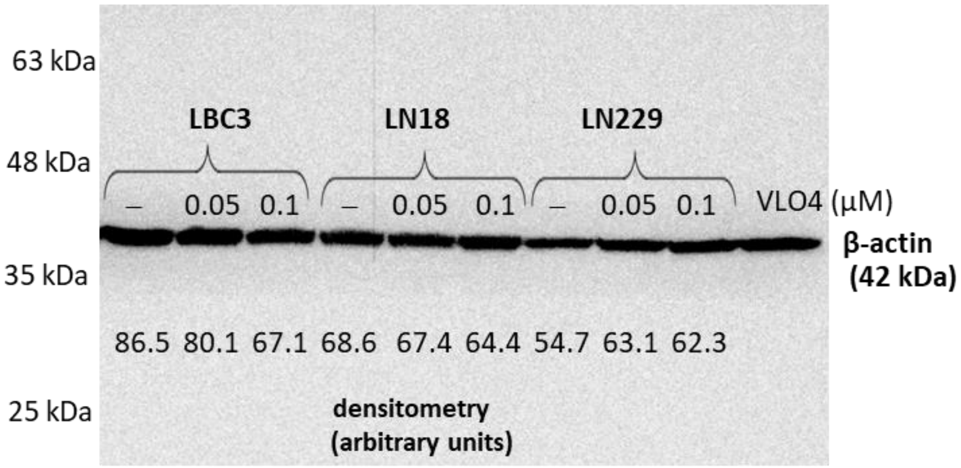

molecular weight marker

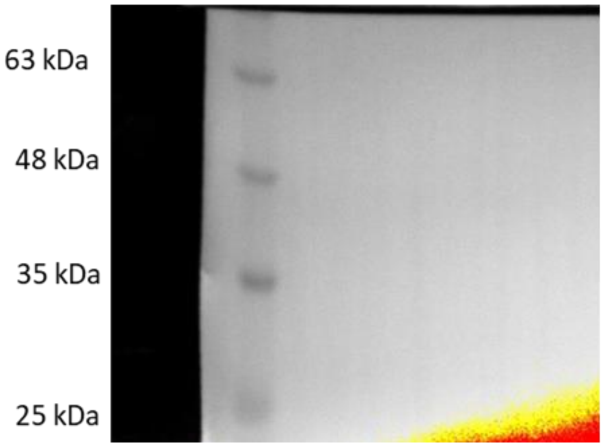

Figure S7. For Figure 8c, Western blot of Bcl2 (A) and β-actin (B) in glioblastoma cell lines LBC3, LN18 and LN229 treated with VLO4 disintegrin. The membranes were cut at a height between the 48 and 75 kDa markers and the lower part of the membrane was used to identify Bcl2 and the upper part for another tested high-molecular protein. Densitometry values of the protein bands and the molecular weight markers are also shown.

(A)

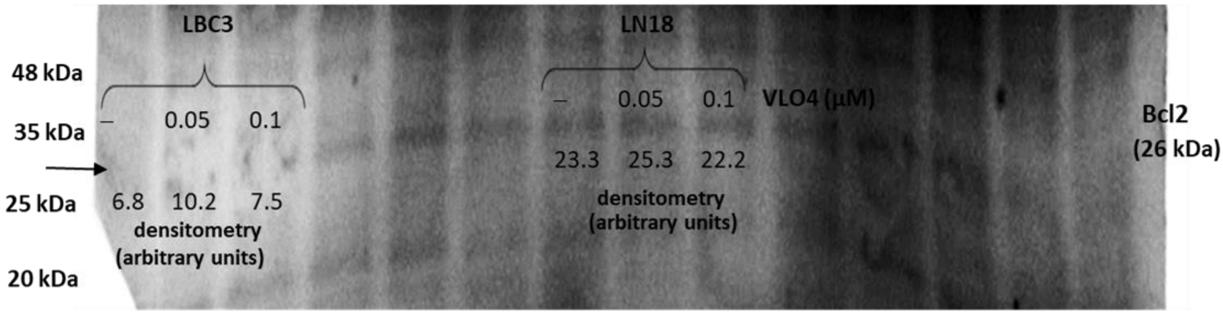

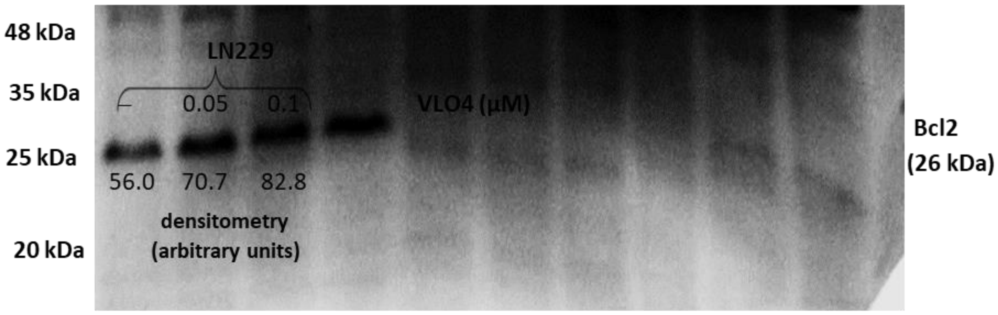

molecular weight marker

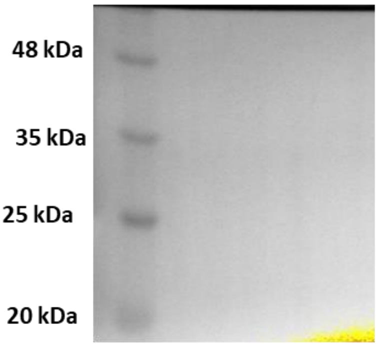

(B)

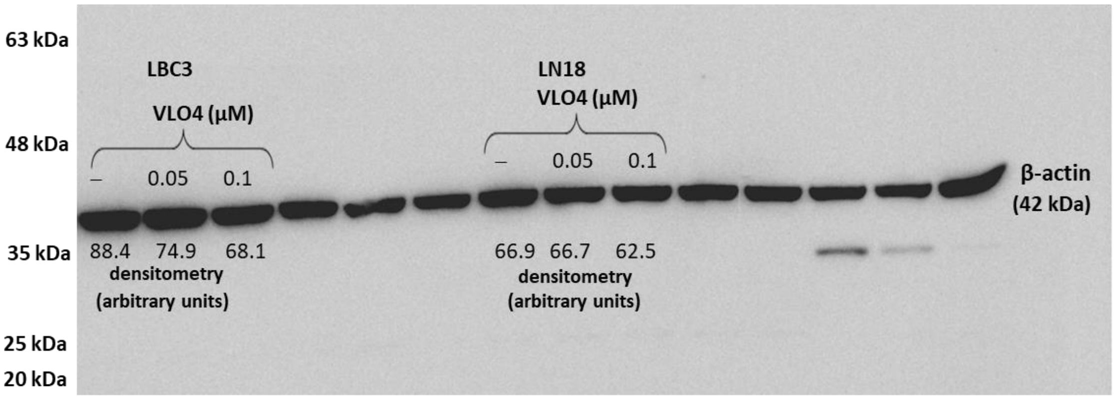

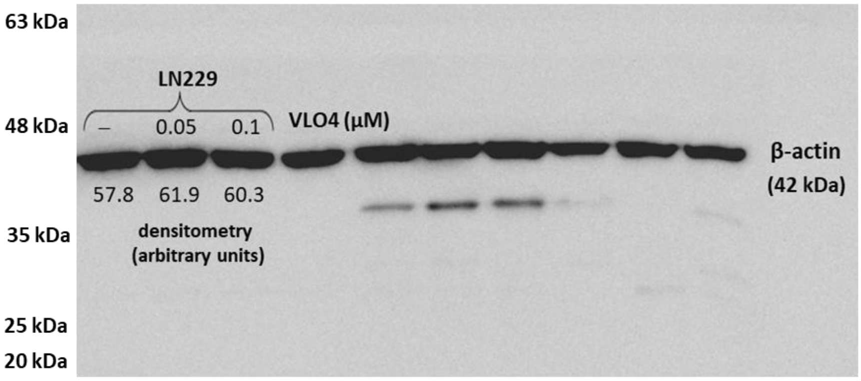

molecular weight marker

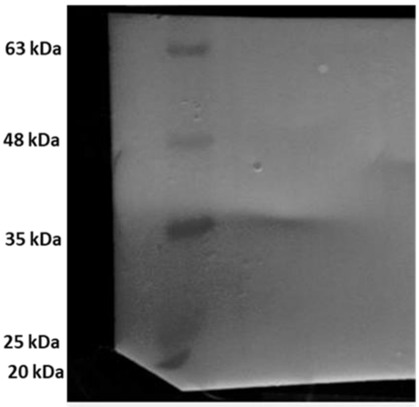

Figure S8. For Figure 8d, Western blot of Bcl-xL (A) and β-actin (B) in glioblastoma cell lines LBC3, LN18 and LN229 treated with VLO4 disintegrin. Densitometry values of the protein bands and the molecular weight markers are shown.

(A)

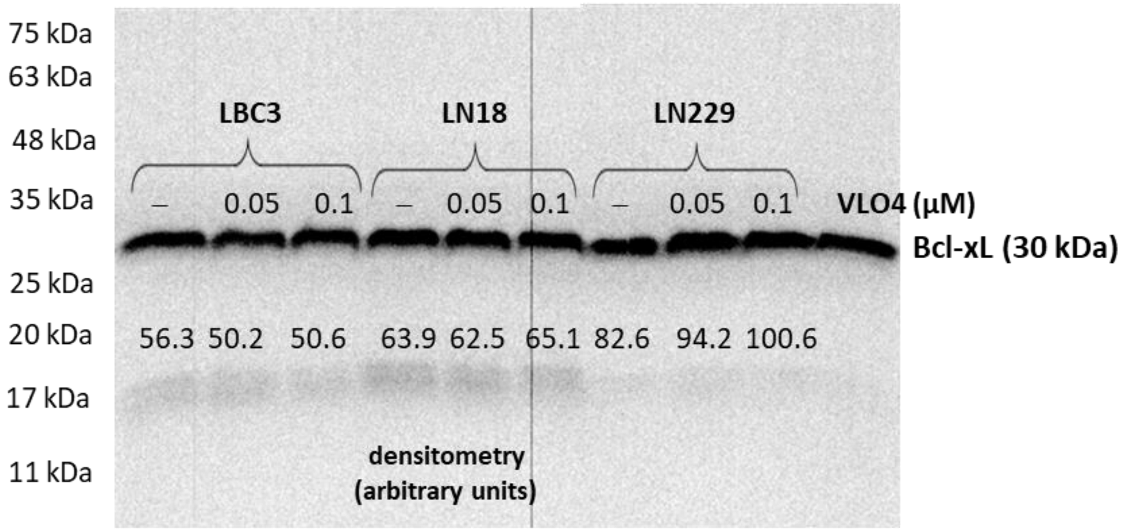

molecular weight marker

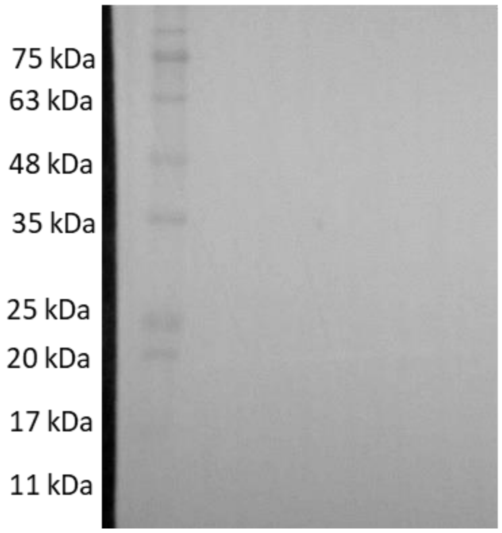

(B)

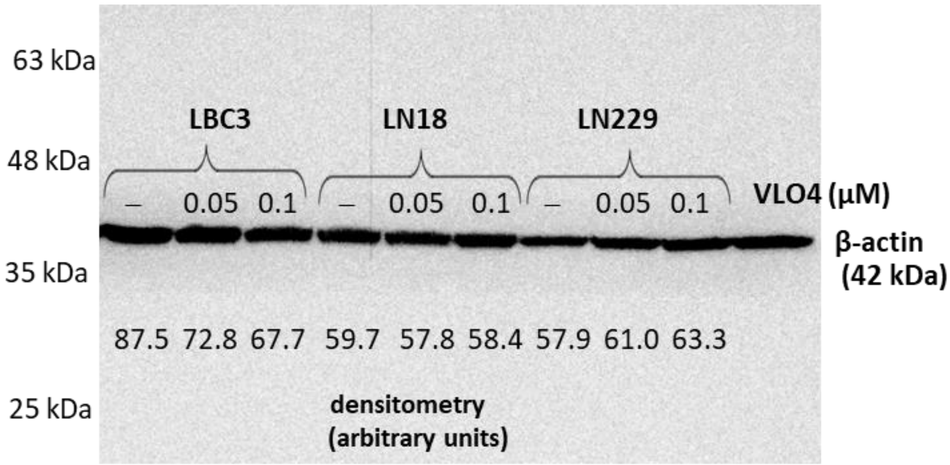

molecular weight marker

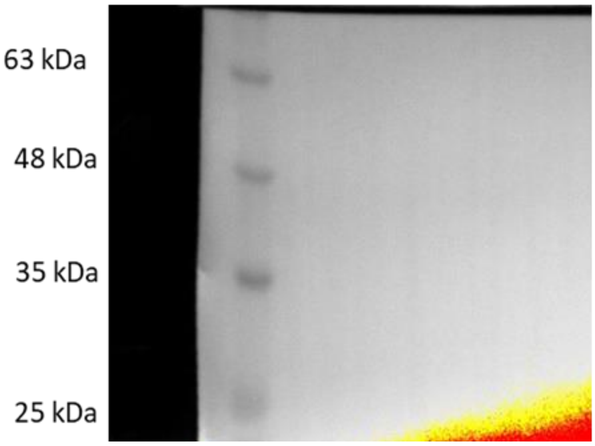

Figure S9. For Figure 6, Gelatin zymography. MMPs were assayed in the medium of glioblastoma cell lines LBC3, LN18 and LN229 treated with VLO4 disintegrin. MMPs are indicated as pro-MMP-2, active MMP-2, active MMP-9 and MMP complexes (130 and 240 kDa). The molecular weight standard (kDa) and densitometry values are shown.

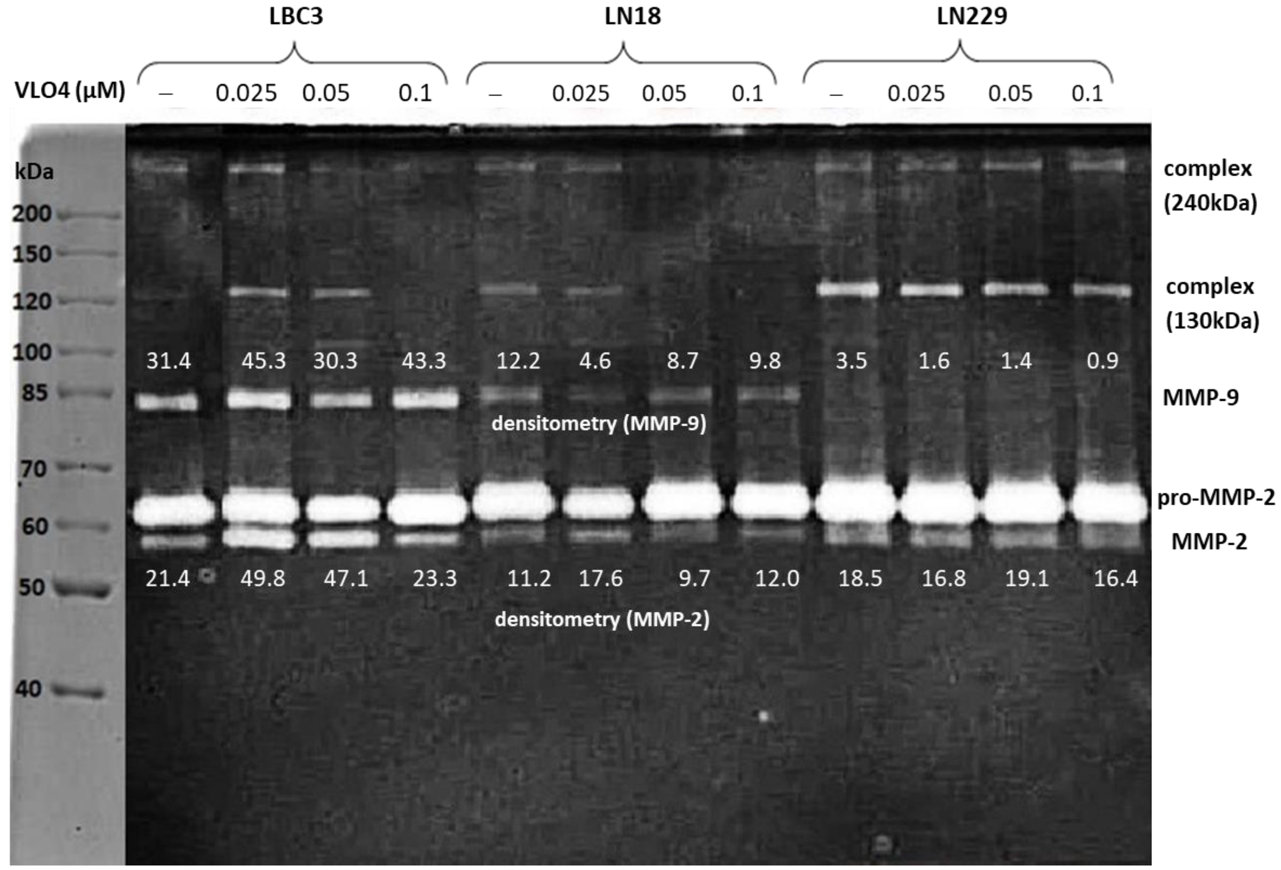

Figure S10. For Figures 1d and 4b, Western blot of integrin subunits.

The molecular weight markers and densitometry values are shown.

Figure 1d **Figure 4b**

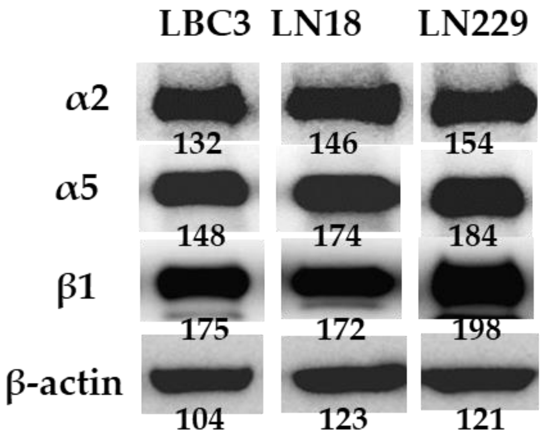

(d)

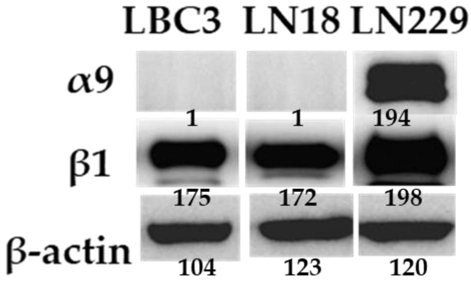

(b)

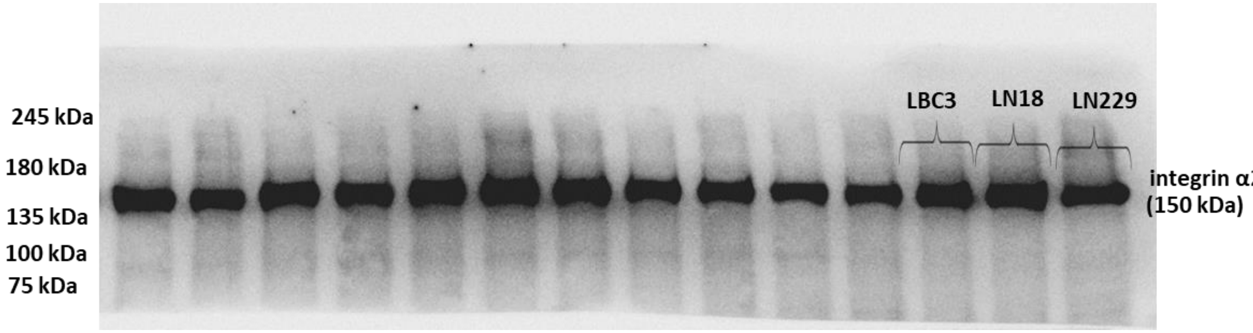

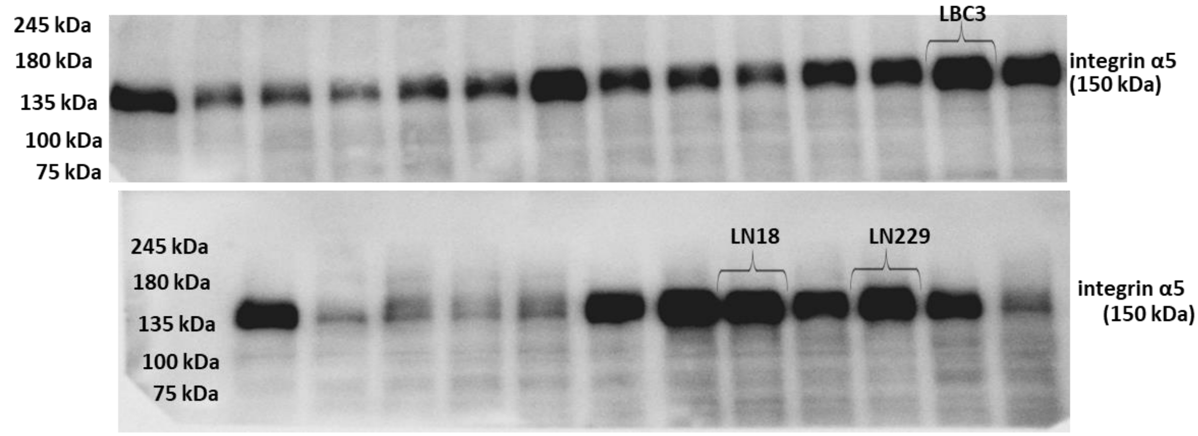

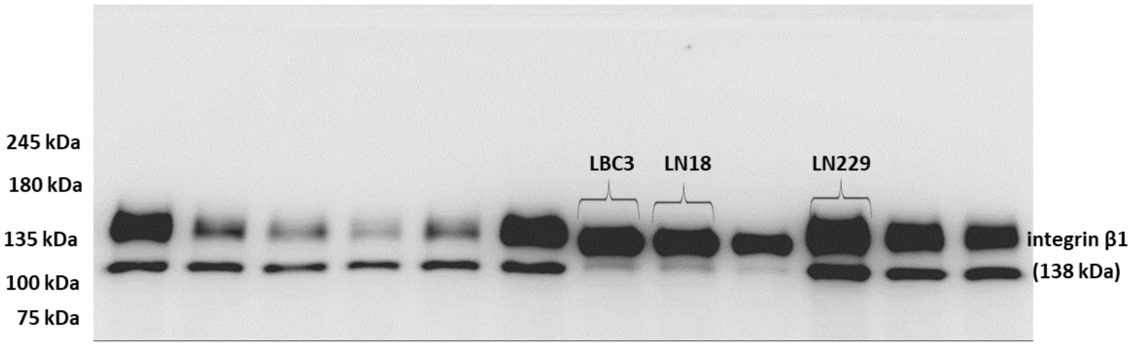

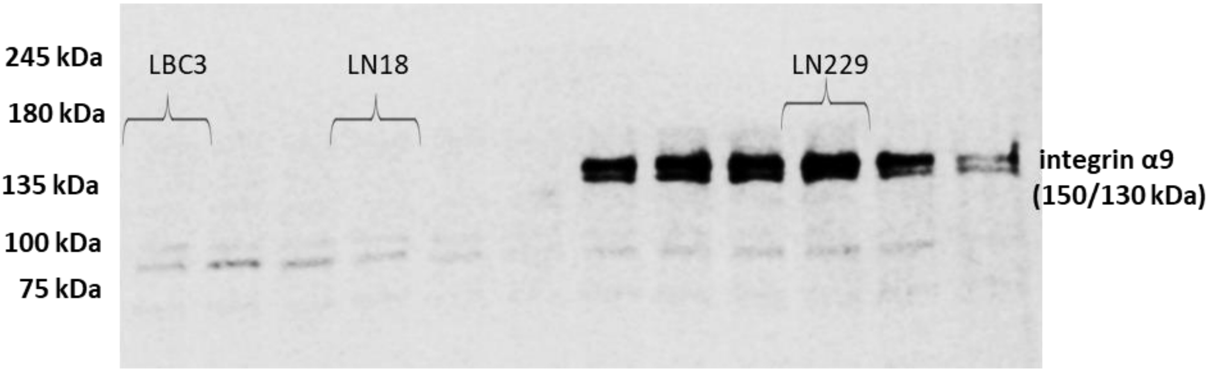

molecular weight marker

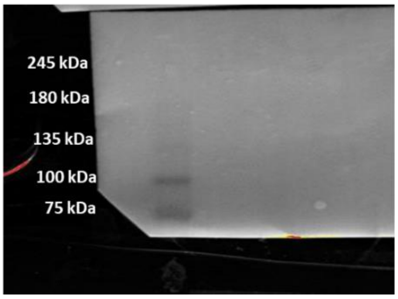

Supplement: Supplementary file 1 [file cancers-15-04805-s001.zip › cancers-2579027-supplementary.pdf]
